# Long-term electricity load forecasting model based on multi-period and multi-scale analysis

**DOI:** 10.1371/journal.pone.0361106

**Published:** 2026-10-30

**Authors:** Xiaolin Zhang, Zhi Liu, Chunyang Wang, Zhen Qian

**Affiliations:** 1 School of Electronic Information Engineering, Changchun University of Science and Technology, Changchun, Jilin, China; 2 Yahe School of Design and Engineering, Haikou University of Economics, Haikou, Hainan, China; 3 Tsinghua University, Beijing, China; 4 Beijing Jiaotong University, Beijing, China; Indiana University Purdue University Indianapolis (IUPUI), UNITED STATES OF AMERICA

## Abstract

Existing long-term power load forecasting models face significant challenges due to the complexity of non-stationary electricity data, inadequate information representation, and suboptimal prediction accuracy. To address these issues, this study introduces PowerNet, a novel forecasting framework based on a multi-period and multi-scale approach. PowerNet first employs Fast Fourier Transform (FFT) to perform multi-period analysis, transforming one-dimensional time series data into two-dimensional (2D) tensors to capture latent periodic patterns. A multi-scale information representation module is then implemented using computer vision-inspired techniques, including Convolutional Neural Networks (CNN), Gated Recurrent Units (GRU), and non-local attention mechanisms to extract robust features from these tensors. Finally, a fully connected layer performs feature reconstruction, while residual connections facilitate multi-step load forecasting. PowerNet was evaluated against state-of-the-art models such as Informer, LogTrans, and ETSformer across two public electricity datasets and one weather dataset. At prediction horizons of 96, 192, 336, and 720 time steps, experimental results demonstrate that PowerNet achieves competitive or superior accuracy on the evaluated datasets. Notably, compared with Autoformer, PowerNet reduces the Mean Absolute Error (MAE) by 18.77% to 37.41% and the Mean Square Error (MSE) by 18.23% to 47.74%, significantly enhancing the reliability of long-term electricity load forecasting.

## Introduction

Against the backdrop of the global decarbonization trend, the transformation of power systems has emerged as a critical task. Load forecasting is an indispensable component of power system planning, as accurate predictions provide a reliable decision-making basis for both system operation and planning. Consequently, research on power load forecasting has become increasingly vital. In particular, enhancing the precision of long-term load forecasting holds significant implications for formulating generation and transmission plans, load switching, and infrastructure development decisions.

Time-series forecasting plays a pivotal role in decision-making across various critical domains, including transportation, medicine, economics, and energy. Currently, power load forecasting methodologies primarily fall into three categories: statistical learning, machine learning, and deep learning.

Statistical learning methods for power load forecasting include autoregressive models [1], Kalman filtering [2], and exponential smoothing [3]. Among these, the Autoregressive Moving Average (ARIMA) model [4] has long been extensively applied in various fields of time-series forecasting. However, the success of the ARIMA model is predicated on the assumption of stationarity, which modern practical power load data often fails to satisfy. Furthermore, ARIMA is typically limited to capturing linear relationships and seasonal patterns, resulting in poor performance when dealing with non-linear relationships and complex time-series patterns. Consequently, the application of ARIMA is constrained by data characteristics, offering limited versatility in the current data-driven era.

Numerous models based on machine learning algorithms have also been applied to power load forecasting. Common algorithms include decision trees [5], Support Vector Machines (SVM) [6], and ensemble learning [7]. Although these methods can capture the non-linear correlations within power load data, they often fail to fully exploit and utilize the inherent temporal information.

With the rapid advancement of deep learning, researchers have found that deep-learning-based methods exhibit robust capabilities in feature mining, non-linear mapping, and self-adaptation. Compared to machine learning, these methods are more suitable for complex time-series modeling and better align with current intricate power load data. However, the characteristics of Convolutional Neural Networks (CNN) [8] are more appropriate for the spatially correlated domain of computer vision rather than time-series forecasting [9,10]. Consequently, Recurrent Neural Networks (RNN) [11] emerged, with numerous studies demonstrating their significant advantages in temporal prediction. Nevertheless, traditional RNNs suffer from vanishing and exploding gradient issues, rendering them ineffective in capturing long-term dependencies.Long Short-Term Memory (LSTM) [12] and Gated Recurrent Units (GRU) [13] were proposed to address these deficiencies [14,15]. Following this, many models based on LSTM and GRU have been applied to load forecasting [16–18]. For instance, Shi et al. [19] combined LSTM with self-attention for short-term forecasting, and Lai et al. [20] designed a hybrid GRU-CNN model for building load demand. Nonetheless, in long-term forecasting tasks, as the input sequence grows, GRU and LSTM may encounter issues of information loss and sparsity of historical data, indicating that long-term dependencies remain difficult to capture even with these architectures [14,21].

In recent years, the Transformer has been widely applied across multiple domains and utilized for long-term time-series forecasting tasks. The model leverages its unique attention mechanism to learn relevant temporal features, possessing a superior ability to capture long-range correlations. Based on the Transformer, Informer model [22] was proposed, which introduces a novel ProbSparse self-attention mechanism to reduce complexity and improve the computational and storage efficiency of the original architecture. Conversely, Zeng et al. [23] expresses skepticism regarding the role of Transformers in long-term forecasting, arguing that such models inevitably suffer from information loss due to the inherent properties of the self-attention mechanism.

Wu et al. [24] proposed TimesNet, which transforms one-dimensional time series into two-dimensional tensors according to dominant periods identified in the frequency domain. This design provides an effective way to represent temporal variations from both intra-period and inter-period perspectives and has demonstrated the potential of introducing computer vision techniques into time-series forecasting. However, the effectiveness of such 2D temporal representations depends not only on the transformation itself, but also on how the reconstructed periodic structures are further modeled. Existing 2D transformation-based methods mainly rely on convolutional feature extraction, which is effective for local temporal variations but may be insufficient for capturing long-range temporal dependencies and globally distributed correlations in long-term electricity load forecasting.

To address this limitation, this paper proposes PowerNet, a multi-period and multi-scale forecasting framework designed for long-term electricity load forecasting. Rather than treating the FFT-based 1D-to-2D transformation as the primary novelty, PowerNet focuses on enhancing representation learning after periodic reconstruction. Specifically, PowerNet introduces a dual-branch information representation module that combines a multi-scale dilated Inception variant with a GRU-based non-local attention branch. The Inception variant captures local intra-period and inter-period variations at different receptive fields, while the GRU-NLA branch models sequential transitions and global temporal correlations. Through multiplicative feature interaction, PowerNet enables local multi-scale features and long-range dependency features to complement each other, thereby improving the representation capacity for complex long-term load forecasting. The primary contributions of this paper are as follows:

1. We propose PowerNet, a multi-period and multi-scale representation framework for long-term electricity load forecasting. Different from existing 2D transformation-based forecasting methods that mainly emphasize periodic restructuring and local convolutional modeling, PowerNet focuses on enhancing feature representation after periodic 2D reconstruction by jointly modeling local multi-scale variations and long-range temporal dependencies.
2. We design a multi-scale dilated Inception variant to extract intra-period and inter-period temporal variations from reconstructed 2D tensors. By using convolutional kernels with different receptive fields and dilation patterns, this branch captures local periodic structures at multiple temporal scales while maintaining computational efficiency.
3. We introduce a GRU-based non-local attention branch to complement the convolutional branch. The GRU component models sequential transitions in load patterns, while the non-local attention mechanism captures global correlations between distant temporal positions. This branch is particularly useful for long-term forecasting scenarios where relevant dependencies may span multiple periods.
4. We develop a multiplicative feature interaction strategy to integrate the Inception variant and GRU-NLA branches. Instead of simply stacking multiple modules, this design allows long-range dependency features to modulate local multi-scale features, thereby improving the robustness of the learned representation.
5. We evaluate PowerNet on two public electricity-related datasets and one meteorological dataset under multiple prediction horizons. The results show that PowerNet achieves competitive or superior forecasting performance compared with representative long-term time-series forecasting baselines, while additional ablation analyses further verify the contribution of its key components.

## Problem definition

Generally, power load forecasting can be categorized into ultra-short-term, short-term, medium-term, and long-term. This paper focuses on long-term power load forecasting (LTPF). In the context of LTPF, the input is defined as a historical power load time series $\left[X^{0}\;,X^{1}...\;,X^{T-1}\right]$ of length *T*, where the superscript denotes the sequence length (i.e., the number of time steps). Each time step data $X_{1D}^{0}\in {\mathbb{R}}^{T\times C}$ contains values for *C* variables. Let the power load sequence for the future *I* time steps be denoted as $\left[{\hat{X}}^{T}\;,{\hat{X}}^{T+1}\;,...,{\hat{X}}^{T+I-1}\right]$. The objective of LTPF is to train a mapping function *f* from the historical time series to the future time series, where the value of *I* is significantly larger than the forecasting horizon typically used in short-term power load forecasting:


$$\left[X^{0}\;,X^{1}...\;,X^{T-1}]\overset{\;f\;\;}{\to }[{\hat{X}}^{T}\;,{\hat{X}}^{T+1}\;,...,{\hat{X}}^{T+I-1}\right]$$

## The PowerNet model

The overall architecture of the proposed long-term power load forecasting model, PowerNet, is illustrated in Fig 1. The model adopts a modular framework designed to address varying temporal patterns across different periods. Specifically, this study proposes a TimesBlock to capture corresponding intra-period and inter-period variations from a multi-scale perspective. Each TimesBlock consists of two primary components: a data processing module and an information representation module.

**Fig 1:**
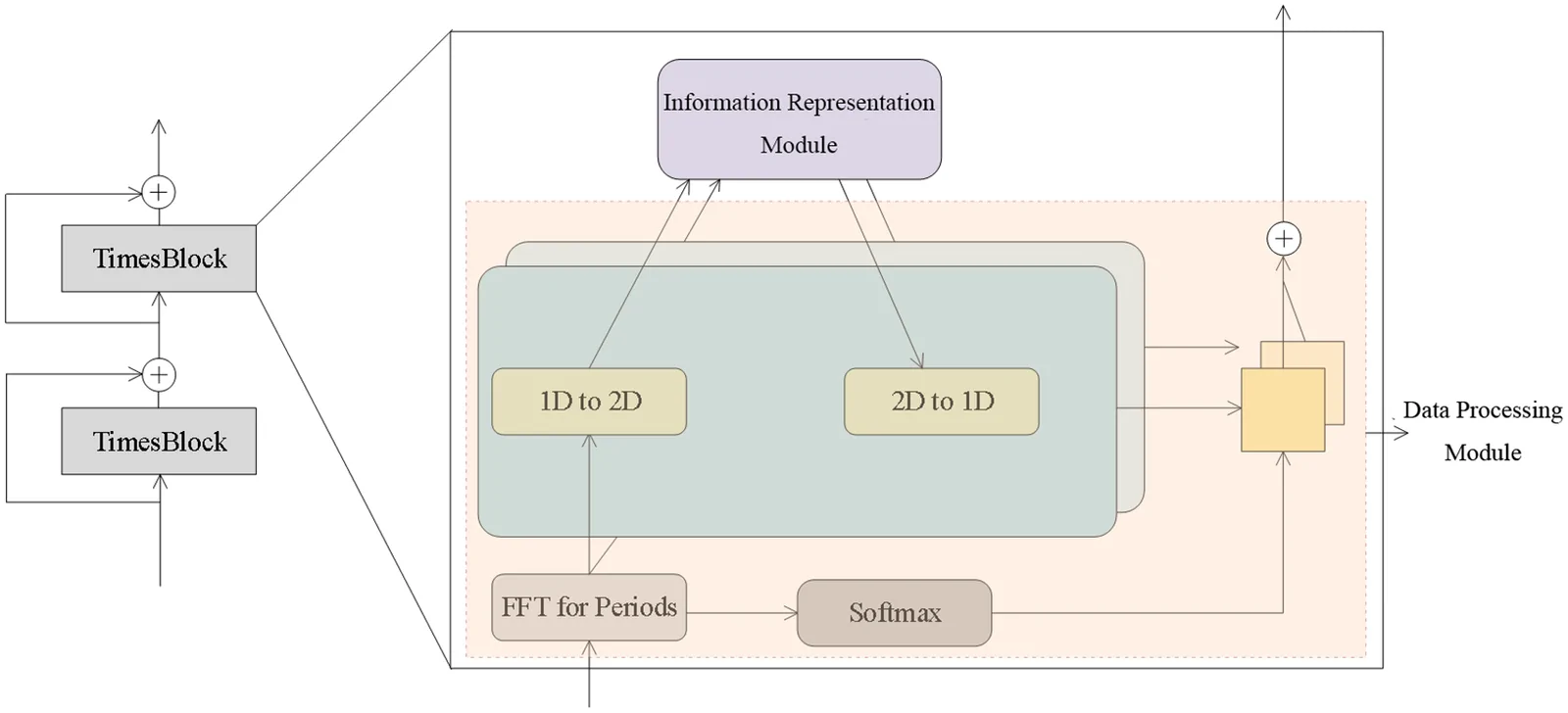
Overall architecture of PowerNet. The diagram illustrates the modular framework including the TimesBlock layers and the multi-scale information representation process.

The model is constructed with two layers of TimesBlocks integrated via residual connections. The input data is denoted as $X_{1D}^{0}\in {\mathbb{R}}^{T\times C}$, representing a time series with *C* variables and a length of *T*. The operational process is defined as follows:


$$\begin{array}{cc} X_{1D}^{1} & =TimesBlock(X_{1D}^{0})+X_{1D}^{0}\\ X_{1D}^{2} & =TimesBlock(X_{1D}^{1})+X_{1D}^{1} \end{array}$$ (1)

### Data processing module

Most existing power load forecasting methods focus predominantly on the temporal dimension of time series to capture sequential dependencies. However, in practical scenarios, time series often manifest diverse patterns—such as heterogeneous periodicities and varying trends—that are intricately intertwined. When modeling the temporal dimension of the raw sequence directly, the underlying temporal relationships are frequently obscured by these complex patterns, making them difficult to capture accurately.

Considering that real-world power loads typically fluctuate with time and seasons, power load data inherently exhibits multi-periodicity, such as daily, monthly, quarterly, and annual cycles. Dependencies exist both within each period (intra-period) and between corresponding time points in adjacent periods (inter-period). To determine the periodicity within the time series, the input 1D time series $X_{1D}^{i}$ (where i∈{0,1,2}) is first analyzed in the frequency domain. Its periodicity is calculated using the Fast Fourier Transform (FFT), as defined in Eq (2):


$$\begin{array}{cc} A & =Avg(Amp(FFT(X_{1D})))\\ \left\{f_{1},...,f_{k}\right\} & =\text{arg}Topk(A),\;f_{*}\in \left\{1,...,\left\lfloor \frac{T}{2}\right\rfloor \right\}\\ p_{i} & =\left\lfloor \frac{T}{f_{i}}\right\rfloor ,\;i\in \{1,...,k\} \end{array}$$ (2)

where FFT(•) and Amp(•) represent the Fast Fourier Transform and the calculation of amplitude values, respectively. $A\in {\mathbb{R}}^{T}$ denotes the amplitude intensity of each frequency in *X*_1*D*_. To mitigate the impact of high-frequency noise, only the *k* frequencies with the highest intensities, $\left\{f_{1},...,f_{k}\right\}$, are selected. Furthermore, considering the conjugate symmetry in the frequency domain, frequencies are selected only within the range {1,...,⌊T/2⌋}.

These selected frequencies correspond to *k* period lengths $\left\{p_{1},...,p_{k}\right\}$. Based on these values, the original 1D time series *X*_1*D*_ is reshaped, a process formulated in Eq (3):


$$\begin{array}{c} X_{2D}^{i}={Reshape}_{p_{i},f_{i}}(Padding(X_{1D})),\;i\in \{1,...,k\} \end{array}$$ (3)

where the length of the time series is not divisible by $p_{i}$, a Padding(•) operation is employed to append zeros at the end of the sequence. This yields a set of 2D tensors $\left\{X_{2D}^{1},X_{2D}^{2},...,X_{2D}^{k}\right\}$, where columns and rows represent intra-period and inter-period variations, respectively. Consequently, these 2D tensors exhibit 2D locality and can be treated as a set of images, facilitating the integration of computer vision techniques for feature extraction.

The workflow of the data processing module is illustrated in Fig 2.

**Fig 2:**
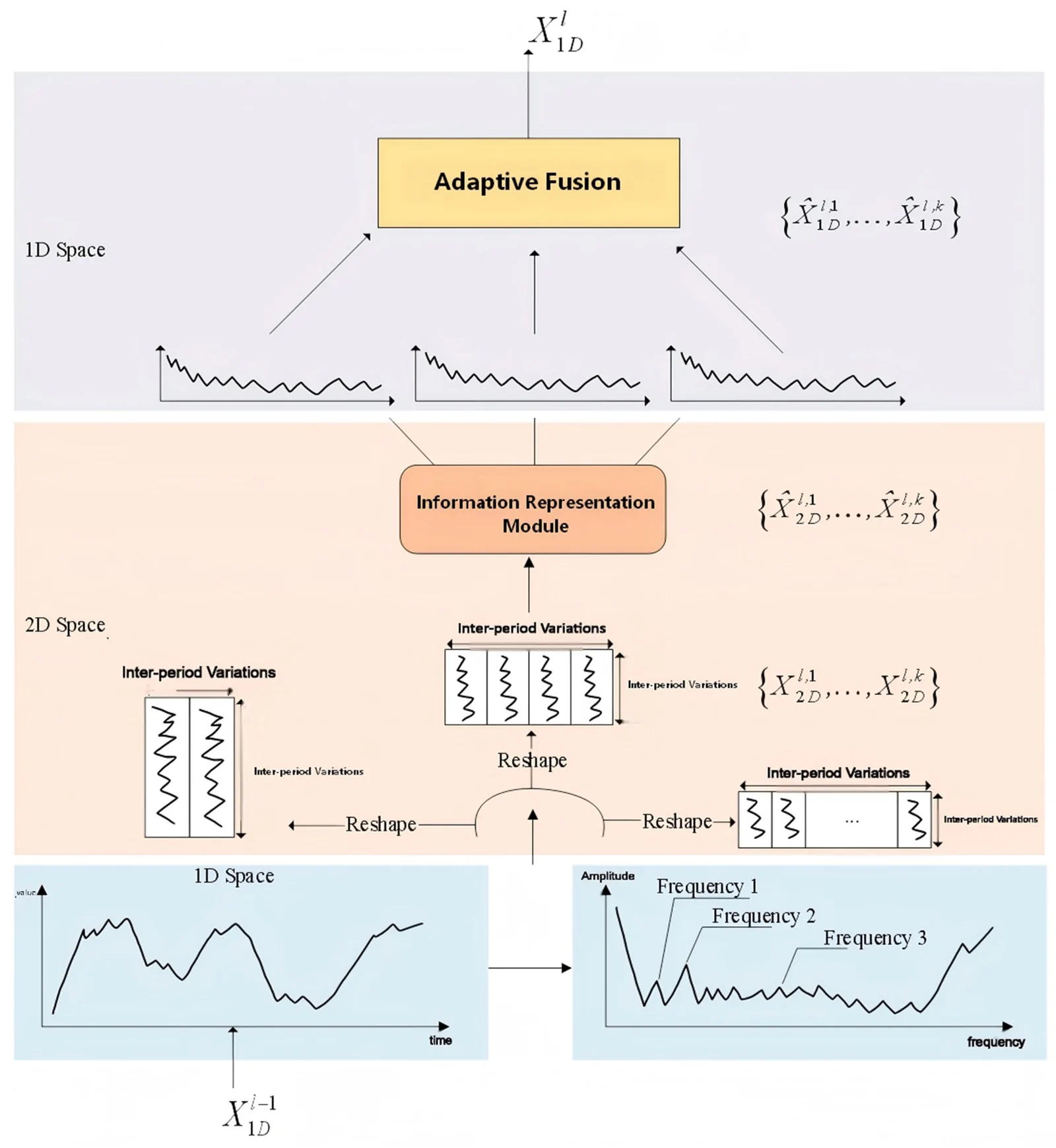
Flowchart of the data processing module. The diagram illustrates the four-step process: (1) Period extraction via FFT and 1D-to-2D transformation; (2) Multi-scale feature representation; (3) 2D-to-1D reshaping; (4) Adaptive fusion of temporal features.

The process primarily consists of the following four steps:

1. The *k* most dominant periods are extracted from the input raw 1D load time series via FFT. The 1D sequence is then partitioned according to these multiple periods and transformed into multiple 2D tensors, as formulated in Eq (2) and Eq (3).
2. The 2D tensors are fed into the information representation module to undergo feature representation, yielding 2D temporal variation features.
3. The resulting features are reshaped back into the 1D domain.
4. The 1D features undergo adaptive fusion [25], where a weighted summation is calculated according to the frequency intensities to produce the final output.

### Information representation module

While the baseline TimesNet model provides a foundation for periodic analysis, it is prone to overfitting in long-term time series forecasting tasks. This vulnerability stems from its reliance on a single Inception block for feature extraction. In long-term electricity load forecasting, data exhibit extensive temporal dependencies; however, TimesNet primarily focuses on local features, resulting in an insufficient capacity to capture non-local relationships and long-term trends within complex sequences.

To address these limitations, a novel information representation module is proposed. The overall architecture of this module is illustrated in Fig 3. The module consists of two parallel feature extraction branches: an Inception variant and a GRU-NLA (Gated Recurrent Unit with Non-Local Attention) layer. The input 2D tensors enter these two branches simultaneously, and their respective outputs are combined via element-wise multiplication to produce the final representation. This multiplication operation effectively enhances the model’s feature representation capacity and generalization ability, thereby mitigating the risk of overfitting.

**Fig 3:**
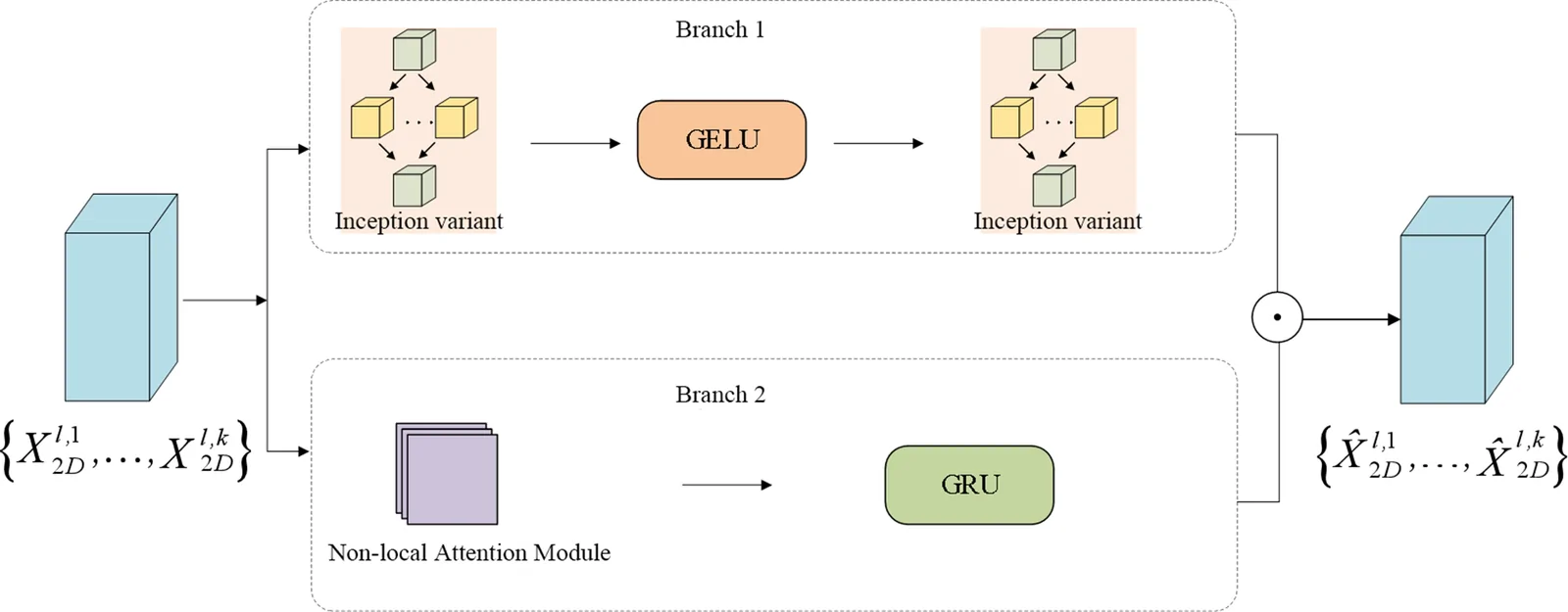
Architecture of the information representation module. The module utilizes a dual-branch structure comprising an Inception variant and a GRU-NLA layer. The final output is generated through the element-wise multiplication of features from both branches to capture both local and long-term dependencies.

#### Inception variant.

As illustrated in Fig 3, Branch 1 is constructed by stacking two Inception variants. To improve gradient flow and mitigate potential vanishing gradient issues, the Gaussian Error Linear Unit (GELU) activation function is incorporated between these two stages.

The Inception variant architecture utilizes multiple parallel convolutional branches, each employing dilated convolutions across several spatial scales. This design enables the network to capture multi-scale feature dynamics within periods (intra-period) and across identical periods (inter-period) of power load data at varying levels of abstraction. The inherent characteristics of dilated convolutions allow the model to expand its receptive field and handle long-term fluctuations more effectively without a significant increase in parameter count, thereby enhancing both forecasting performance and generalization. Furthermore, the module automatically learns the optimal weights for each branch. By performing a weighted average based on these learned relationships, the outputs are effectively fused to strengthen the model’s representational power.

In addition, each convolutional kernel is followed by a Batch Normalization (BN) layer. These BN layers normalize the outputs, facilitating faster model convergence, improving stability, and further addressing gradient-related training difficulties. The specific architecture of the Inception variant is depicted in Fig 4.

**Fig 4:**
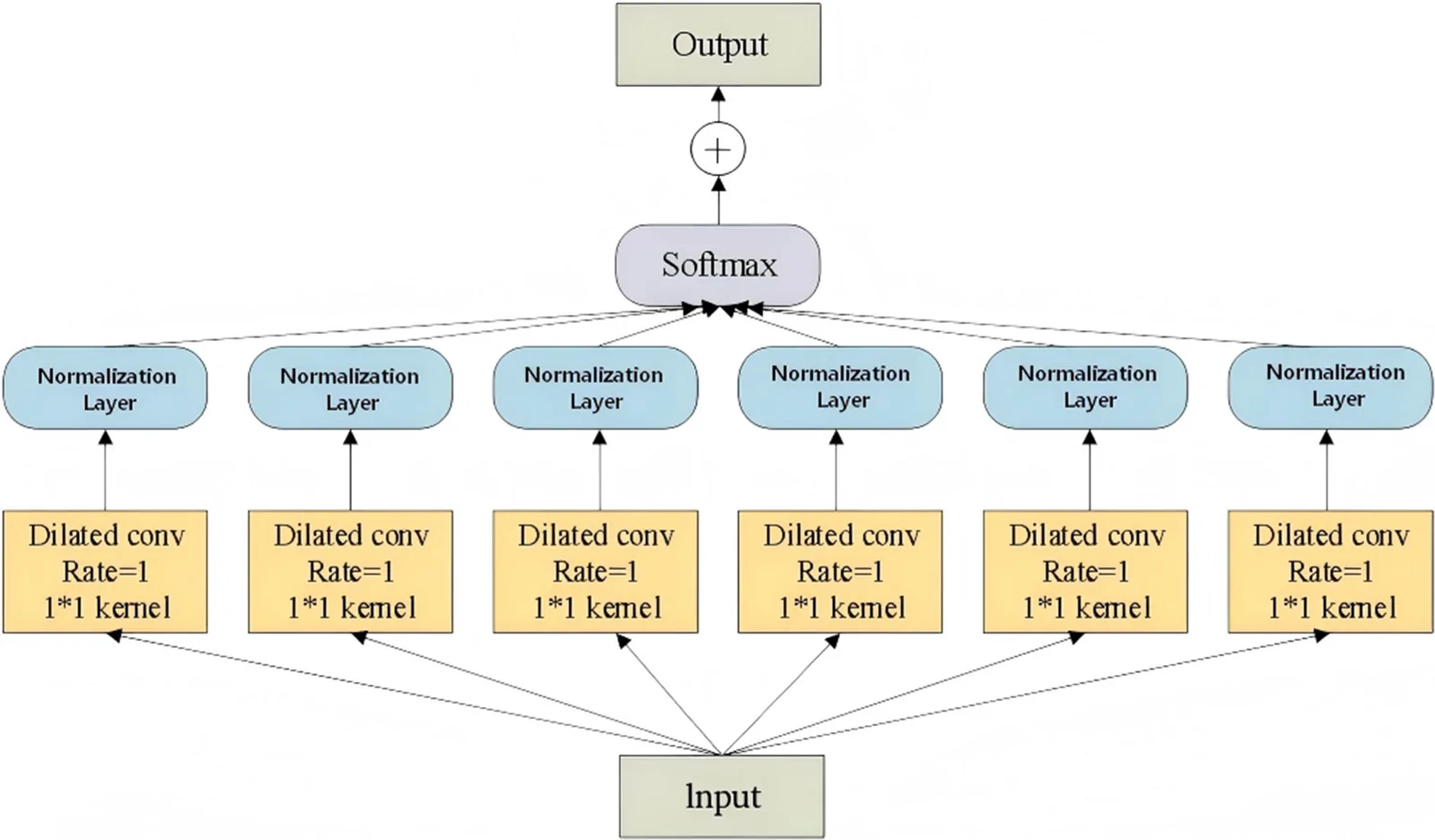
Architecture of the Inception variant. The diagram shows the parallel convolutional branches utilizing multi-scale dilated convolutions, followed by Batch Normalization (BN) and Gaussian Error Linear Unit (GELU) activation to facilitate multi-scale feature fusion.

#### Non-local attention mechanism.

In the Inception branch, local connections within the sub-network are utilized between two Inception variant layers to capture the local features of the 2D power load. Subsequently, global information is obtained through the residual connections between two TimesBlock layers in the overall network, thereby expanding the receptive field of the higher-level network.

However, during the processes of data sampling and layer-by-layer transmission, traditional Convolutional Neural Networks (CNNs) may suffer from significant information loss, which imposes limitations on acquiring global information and modeling long-term dependencies. In long-term power load forecasting, variations within and across periods are typically highly correlated and globally distributed. Conventional convolution operations may be insufficient to capture these global correlations between distant data points.

The non-local attention mechanism addresses these challenges by performing weighted calculations across the intra-period and inter-period variations within the 2D temporal tensors, a methodology consistent with recent advances in global dependency modeling. This approach overcomes the inherent focus of CNNs on local features. The architecture of the non-local attention mechanism is illustrated in Fig 5. The input data shape is C×H×W, where *C* denotes the number of input channels, *H* represents the length of a single period, and *W* is the number of periods (H×W corresponds to the total sequence length). The architecture comprises four parallel paths: the top two paths compute correlations between variables; the third path integrates these correlations into the feature map via matrix multiplication; and the final path performs feature fusion across different stages.

**Fig 5:**
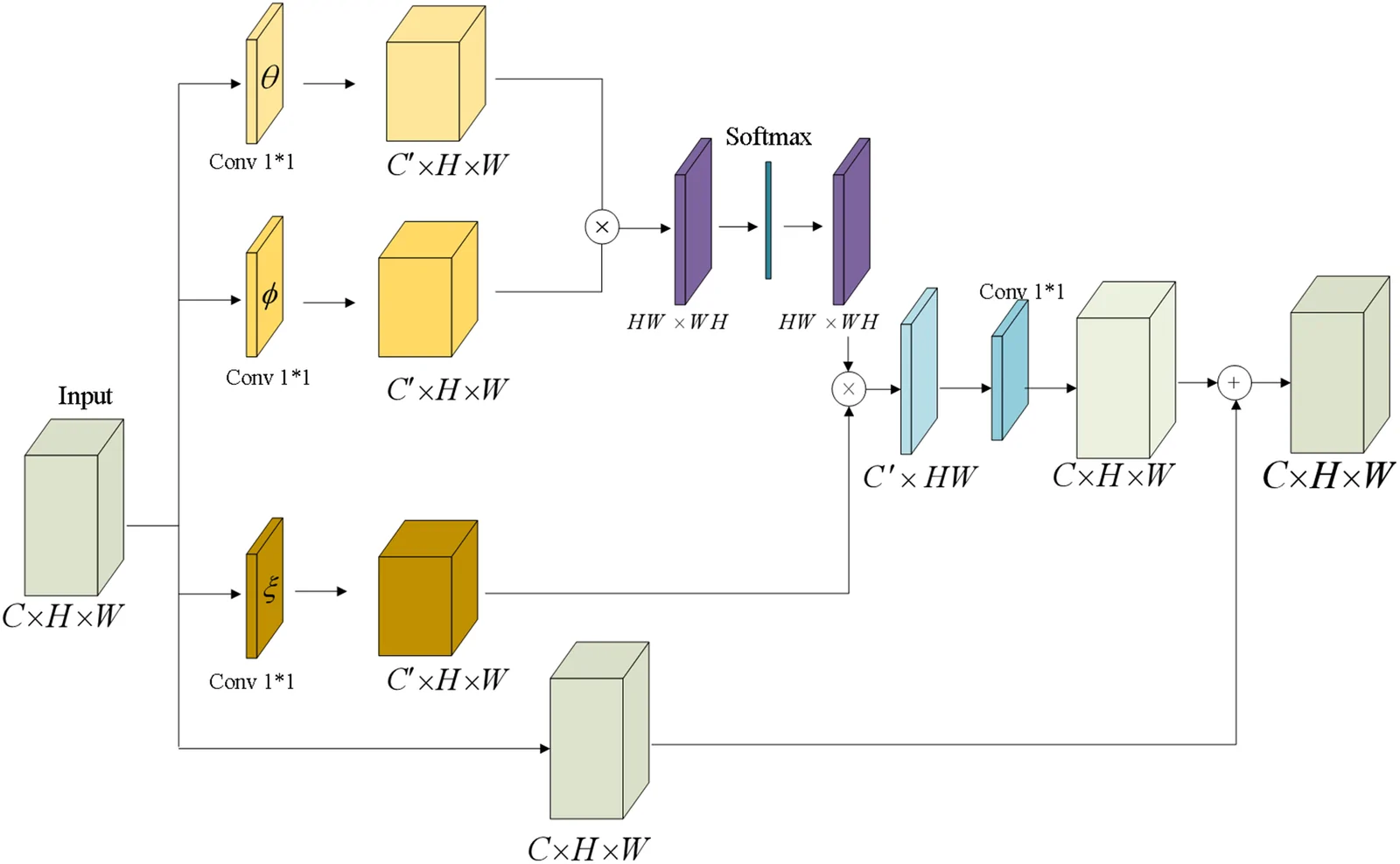
Architecture of the non-local attention module. The diagram illustrates the four-path processing structure, including correlation computation, matrix multiplication for feature integration, and final stage fusion.

The mathematical formulation of the non-local attention mechanism is defined in Eq (4):


$$\begin{array}{c} y_{i}=\frac{1}{C(x)}\sum_{\forall j}f(x_{i},x_{j})\;\xi (x_{j}) \end{array}$$ (4)

where *x* is the input data, $x_{i}$ represents a vector with the same dimension as the number of channels, and $y_{i}$ is the output vector. The indices *i* and *j* denote positions within the input data. The function $f(x_{i},x_{j})$ evaluates the similarity between positions *i* and *j*, yielding a normalized weight that reflects their correlation. The term $\xi (x_{j})$ computes the feature representation at position *j*, while *C*(*x*) denotes the normalization factor.

Consequently, this non-local attention module can be effectively integrated into the proposed model. By incorporating this mechanism, the model acquires more comprehensive global information regarding load variations and captures long-term dependencies, thereby enhancing the accuracy of long-term power load forecasting.

#### Gated recurrent unit.

To address the issues of excessive parameters and structural complexity in LSTM modules, which can lead to overfitting, a variant known as the Gated Recurrent Unit (GRU) was proposed [13]. While each Recurrent Neural Network (RNN) variant is composed of replicated instances of a specific module, the GRU simplifies this structure compared to LSTM. As illustrated in Fig 6, a GRU neuron comprises only two gates: the update gate ($z_{t}$) and the reset gate ($r_{t}$).

**Fig 6:**
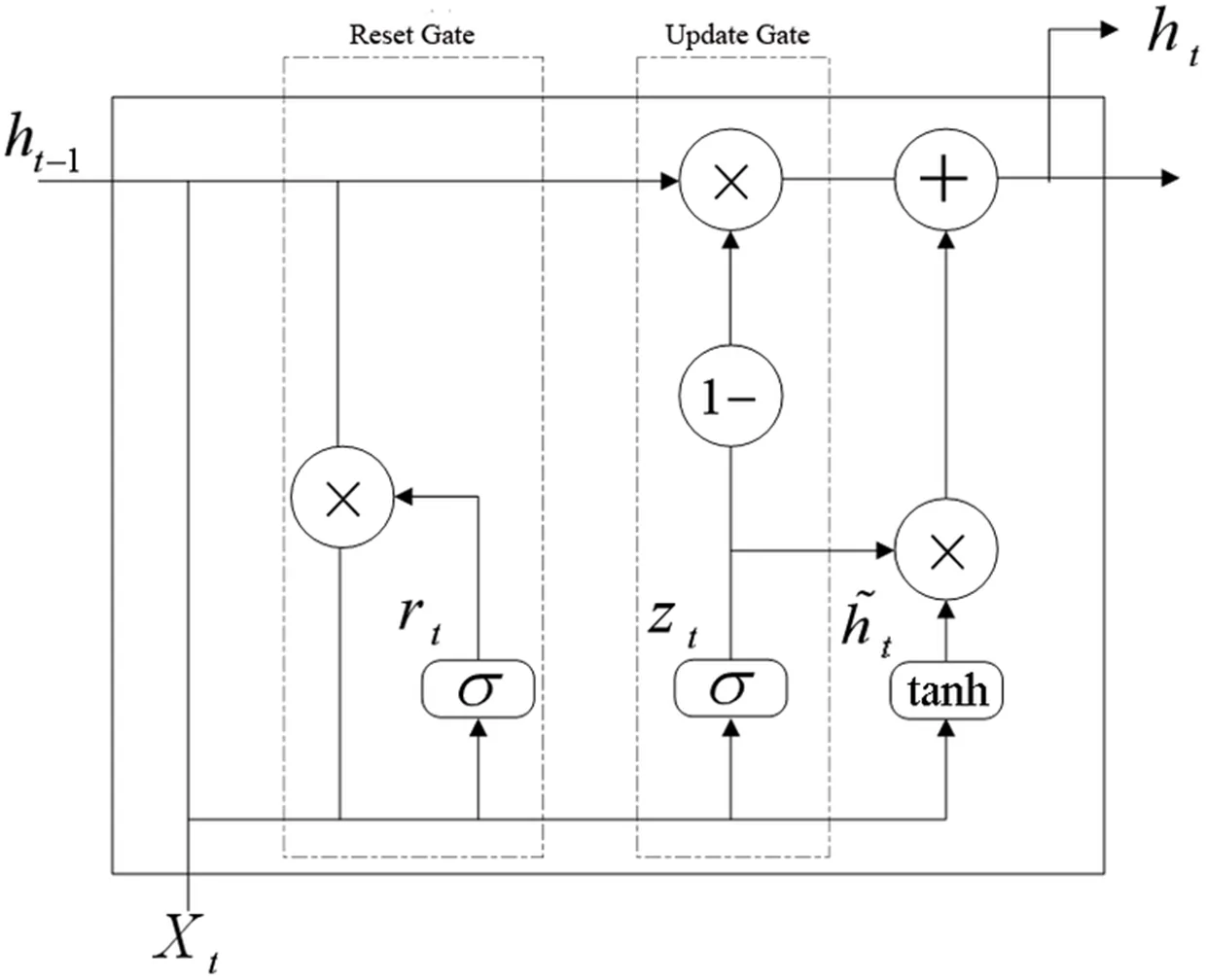
Architecture of the GRU module. The diagram illustrates the internal structure of the GRU cell, highlighting the reset and update gates that regulate information flow and hidden state updates.

The calculation for the reset gate is defined as follows:


$$\begin{array}{c} r_{t}=\sigma (W_{r}\cdot [h_{t-1},X_{t}]+b_{r}) \end{array}$$ (5)

where $\left[h_{t-1},X_{t}\right]$ represents the concatenation of the previous hidden state vector $h_{t-1}$ and the current input vector $X_{t}$. $W_{r}$ and $b_{r}$ denote the weight matrix and the bias term, respectively.

The update gate elements are computed using a similar mechanism as in Eq (5), utilizing distinct weights and biases:


$$\begin{array}{c} z_{t}=\sigma (W_{z}\cdot [h_{t-1},X_{t}]+b_{z}) \end{array}$$ (6)

Subsequently, the candidate hidden state ${\tilde{h}}_{t}$ is calculated according to the information flow depicted in Fig 6:


$$\begin{array}{c} {\tilde{h}}_{t}=\tanh(W_{\tilde{h}}\cdot [r_{t}⊗h_{t-1},X_{t}]+b_{\tilde{h}}) \end{array}$$ (7)

Finally, the hidden state vector at the current time step *t* is determined by combining the previous hidden state, the update gate, and the candidate hidden state:


$$\begin{array}{c} h_{t}=(1-z_{t})⊗h_{t-1}+z_{t}⊗{\tilde{h}}_{t} \end{array}$$ (8)

Through the operations defined in Eq (5) to Eq (8), the hidden state is iteratively updated across time steps. In power load forecasting, the update gate regulates the transmission of information from the previous state; a higher value indicates greater retention of historical data, aiding the capture of long-term dependencies. Conversely, the reset gate determines how much previous information is ignored, assisting the model in identifying short-term patterns and seasonal features within the electricity consumption sequence.

#### GRU-NLA layer.

The GRU-NLA layer branch plays a pivotal role within the proposed model framework, aiming to compensate for the limitations of the multi-scale dilated convolutional branch. It extracts and processes input features to facilitate more accurate and reliable long-term load forecasting. The integrated architecture of this branch is illustrated within the overall information representation module in Fig 3.

First, the GRU-NLA layer branch integrates a Gated Recurrent Unit (GRU) module. By leveraging GRU units, this module performs in-depth time-series modeling of the load data, thereby effectively capturing the periodicity and trend information inherent in the load profiles.

Secondly, the GRU-NLA layer branch incorporates a Non-local Attention (NLA) module. Within the NLA mechanism of this model, the similarity function *f* is normalized via dot-product operations and the Softmax activation function. Three convolutional layers, denoted as ξ(x), θ(x), and ϕ(x), are employed as neural network mappings, where $W_{\xi }$, $W_{\theta }$, and $W_{\phi }$ represent the respective weight matrices. The specific mathematical formulations are defined as follows:


$$\begin{array}{c} f(x_{i},x_{j})=softmax(\theta (x_{i}{)}^{T}\phi (x_{j})) \end{array}$$ (9)

$$\begin{array}{c} \xi (x_{j})=W_{\xi }x_{j} \end{array}$$ (10)

$$\begin{array}{c} \theta (x_{i})=W_{\theta }x_{i} \end{array}$$ (11)

$$\begin{array}{c} \phi (x_{j})=W_{\phi }x_{j} \end{array}$$ (12)

$$\begin{array}{c} C(x)=\sum_{\forall j}f(x_{i},x_{j}) \end{array}$$ (13)

This attention mechanism is capable of capturing both global and local correlations within the input data, facilitating the identification of latent long-term dependencies in the load profiles. Such a mechanism allows the model to better comprehend the complex interactions among load data points, thereby enhancing the accuracy and stability of the forecasting model. In summary, the GRU-NLA layer branch leverages temporal sequence information and non-local correlations to provide robust feature extraction for long-term power load forecasting tasks.

## Experiments and analysis

### Experimental data

To evaluate the performance of the proposed model, two electricity-related public datasets are employed for comparative experiments: the ECL (Electricity Consumption Load) dataset [24] and the ETT (Electricity Transformer Temperature) dataset [24]. Furthermore, to verify the robustness and generalizability of the model, a public meteorological dataset, WTH (Weather) [24], is also included. The detailed characteristics of these datasets are summarized in Table 1.

**Table 1:** Basic characteristics of the datasets.

Dataset	Data volume	Time interval (min)
ECL	26,704	60
ETTm1	69,680	15
WTH	52,697	10

Data volume refers to the total number of recorded observations. Training, validation, and testing sets are partitioned according to a ratio of 7:2:1.

The ECL dataset covers the hourly electricity consumption (measured in kWh) of 321 clients from 2012 to 2014. Each column in the dataset represents the energy consumption of an individual client. For clients added after 2011, their consumption values prior to creation are set to zero.

The ETT dataset contains two years of power transformer temperature data from a region in China. Specifically, the ETTm1 subset records data at 15-minute intervals, where each data point includes the timestamp, six distinct types of external load values, and the oil temperature.

The WTH dataset comprises meteorological data collected from a location in Germany throughout 2020. Measurements were recorded every 10 minutes, with each entry encompassing 21 meteorological indicators, such as humidity and temperature. In the experiments, the training, validation, and testing sets for all datasets are partitioned according to a ratio of 7:2:1.

### Baseline models and evaluation metrics

Several mainstream deep learning models for long-term time series forecasting are selected as experimental baselines: LSTM [12], Informer [22], Autoformer [25], ETSformer [26], and LogTrans [27]. Specifically, LSTM addresses the vanishing gradient problem to capture long-term dependencies. Informer utilizes ProbSparse self-attention and self-attention distilling modules, while Autoformer introduces a series-correlation mechanism. ETSformer extends the Autoformer architecture by incorporating traditional time-series decomposition for trend extraction, and LogTrans utilizes a LogSparse attention mechanism to enhance efficiency.

To further clarify the motivation of the proposed PowerNet model, Table 2 summarizes the main ideas, advantages, and limitations of the benchmark models. These models represent different technical routes for long-term time-series forecasting, including recurrent temporal modeling, sparse attention, decomposition-based forecasting, and 2D temporal variation modeling. However, most of them focus on a specific aspect of temporal representation. In contrast, PowerNet aims to jointly model periodic structures, local multi-scale variations, sequential transitions, and global temporal correlations within a unified framework.

**Table 2:** Comparison of representative benchmark models for long-term forecasting.

Model	Main idea	Advantages	Limitations
LSTM	Recurrent temporal modeling	Captures sequential dependencies	Limited efficiency for long sequences
Informer	ProbSparse self-attention	Reduces attention complexity	May lose detailed local patterns
LogTrans	LogSparse attention	Improves long-sequence efficiency	Depends on sparse attention structure
Autoformer	Decomposition and auto-correlation	Captures trend and seasonal patterns	Less focused on local multi-scale variations
ETSformer	Exponential smoothing Transformer	Models trend-seasonal structures	Relatively high model complexity
TimesNet	Periodic 2D temporal variation modeling	Captures intra-period and inter-period variations	Relies mainly on convolutional representation
PowerNet	Multi-period and multi-scale representation	Integrates periodic, local, and global modeling	Requires additional component-wise design

To quantify the performance of the proposed PowerNet model, two standard evaluation metrics are adopted: Mean Square Error (MSE) and Mean Absolute Error (MAE). Their respective calculation formulas are defined in Eq (14) and Eq (15):


$$\begin{array}{c} MSE(y,\hat{y})=\frac{1}{m}\sum_{i=1}^{m}(y_{i}-{\hat{y}}_{i}{)}^{2} \end{array}$$ (14)

$$\begin{array}{c} MAE(y,\hat{y})=\frac{1}{m}\sum_{i=1}^{m}|y_{i}-{\hat{y}}_{i}| \end{array}$$ (15)

where *m* represents the number of samples, $y_{i}$ denotes the ground truth value, and ${\hat{y}}_{i}$ is the predicted value. Lower values of MSE and MAE indicate higher prediction accuracy, as the model’s outputs are more closely aligned with the actual values.

### Experimental setup

All experiments were conducted on a server equipped with an Intel Xeon Platinum 8358P CPU and an NVIDIA GeForce RTX 3090 GPU, utilizing the PyTorch deep learning framework. Following standard preprocessing procedures, normalization and positional encoding were applied to all datasets. Each dataset was chronologically partitioned into training, validation, and testing sets with a ratio of 7:2:1. To improve the reliability of the findings, each experiment was repeated over three independent trials. The main comparison table reports the average results, and the revised robustness analysis further reports mean ± standard deviation under representative settings.

In the experiments, the prediction horizons for all datasets were set to {96, 192, 336, 720} with a fixed input sequence length of 96. An early stopping strategy was implemented to prevent overfitting and optimize training efficiency. The specific hyperparameter configurations are summarized in Table 3. In addition, we conducted a lightweight sensitivity analysis on the number of selected dominant periods k, which is the key hyperparameter in the FFT-based periodic transformation.

**Table 3:** Key configuration parameters of the model.

Parameter name	Parameter value
Number of strongest periods *k* in FFT	5
Activation function	GELU
Optimizer	Adam
Initial learning rate	0.0001
Loss function	MSE
Dropout coefficient	0.3
Convolutional kernels in Inception variant	6
Batch size	32

### Comparative experiments

Table 4 presents the performance evaluation results of PowerNet and the comparative baseline models on the test sets. To ensure a fair comparison, all models were evaluated under the same long-term forecasting protocol, including the same chronological data partitioning strategy, input sequence length, prediction horizons, preprocessing procedure, and evaluation metrics. For baseline models, we followed the official implementations or widely used benchmark configurations reported in the corresponding studies, and the recommended hyperparameters were adopted when available. We have revised this description to clarify that the baseline results were obtained under consistent forecasting settings rather than from a different dataset or inconsistent experimental protocol.

**Table 4:** Comparison of prediction performance across three datasets.

Dataset	Horizon	PowerNet		Informer		LogTrans		Autoformer		ETSformer		LSTM	
		MSE	MAE	MSE	MAE	MSE	MAE	MSE	MAE	MSE	MAE	MSE	MAE
ECL	96	0.165	0.269	0.274	0.368	0.258	0.357	0.201	0.357	0.187	0.304	0.375	0.437
	192	0.182	0.285	0.296	0.386	0.266	0.368	0.222	0.334	0.199	0.315	0.442	0.473
	336	0.195	0.298	0.300	0.394	0.280	0.380	0.231	0.338	0.212	0.329	0.439	0.473
	720	0.227	0.322	0.373	0.439	0.283	0.376	0.254	0.361	0.233	0.345	0.980	0.814
	Avg	0.192	0.293	0.311	0.397	0.272	0.370	0.227	0.348	0.208	0.323	0.559	0.549
ETTm1	96	0.322	0.366	0.672	0.571	0.600	0.546	0.505	0.475	0.375	0.398	0.863	0.664
	192	0.376	0.393	0.795	0.669	0.837	0.700	0.553	0.496	0.408	0.410	1.113	0.776
	336	0.411	0.416	1.212	0.871	1.124	0.832	0.621	0.537	0.435	0.428	1.267	0.832
	720	0.483	0.457	1.166	0.823	1.153	0.820	0.671	0.561	0.499	0.462	1.324	0.858
	Avg	0.398	0.408	0.961	0.734	0.929	0.725	0.588	0.517	0.429	0.425	1.142	0.783
WTH	96	0.179	0.233	0.300	0.384	0.458	0.490	0.266	0.336	0.197	0.281	0.369	0.406
	192	0.221	0.266	0.598	0.544	0.658	0.589	0.307	0.367	0.237	0.312	0.416	0.435
	336	0.284	0.307	0.578	0.523	0.797	0.652	0.359	0.395	0.298	0.353	0.455	0.454
	720	0.353	0.357	1.059	0.741	0.869	0.675	0.419	0.428	0.352	0.288	0.535	0.520
	Avg	0.259	0.291	0.634	0.548	0.696	0.602	0.338	0.382	0.271	0.309	0.444	0.454

Overall, the proposed PowerNet model achieves competitive or superior performance compared with the selected baseline models across the three evaluated datasets. On the ECL dataset, compared with the top-performing baseline model (ETSformer), PowerNet achieves an average reduction of 7.69% in MSE and 9.29% in MAE. On the ETTm1 dataset, the average reductions in MSE and MAE are 7.23% and 4.00%, respectively. On the WTH meteorological dataset, after correcting the horizon-720 result, PowerNet achieves average MSE and MAE values of 0.259 and 0.291, respectively, showing competitive overall performance compared with the strongest baseline models.

These results indicate that PowerNet achieves high precision on the evaluated long-term forecasting tasks. Moreover, its performance on the WTH dataset provides preliminary evidence that the proposed framework can be transferred to a non-electricity time-series dataset. However, since the current evaluation is limited to two electricity-related datasets and one weather dataset, broader validation on additional datasets is still required before making stronger claims about general applicability across diverse time-series domains.

Fig 7 illustrates partial average test set results of the proposed model on the ECL dataset. As observed, the model is highly effective in capturing the periodic information inherent in the time series, even as the forecasting horizon increases.

**Fig 7:**
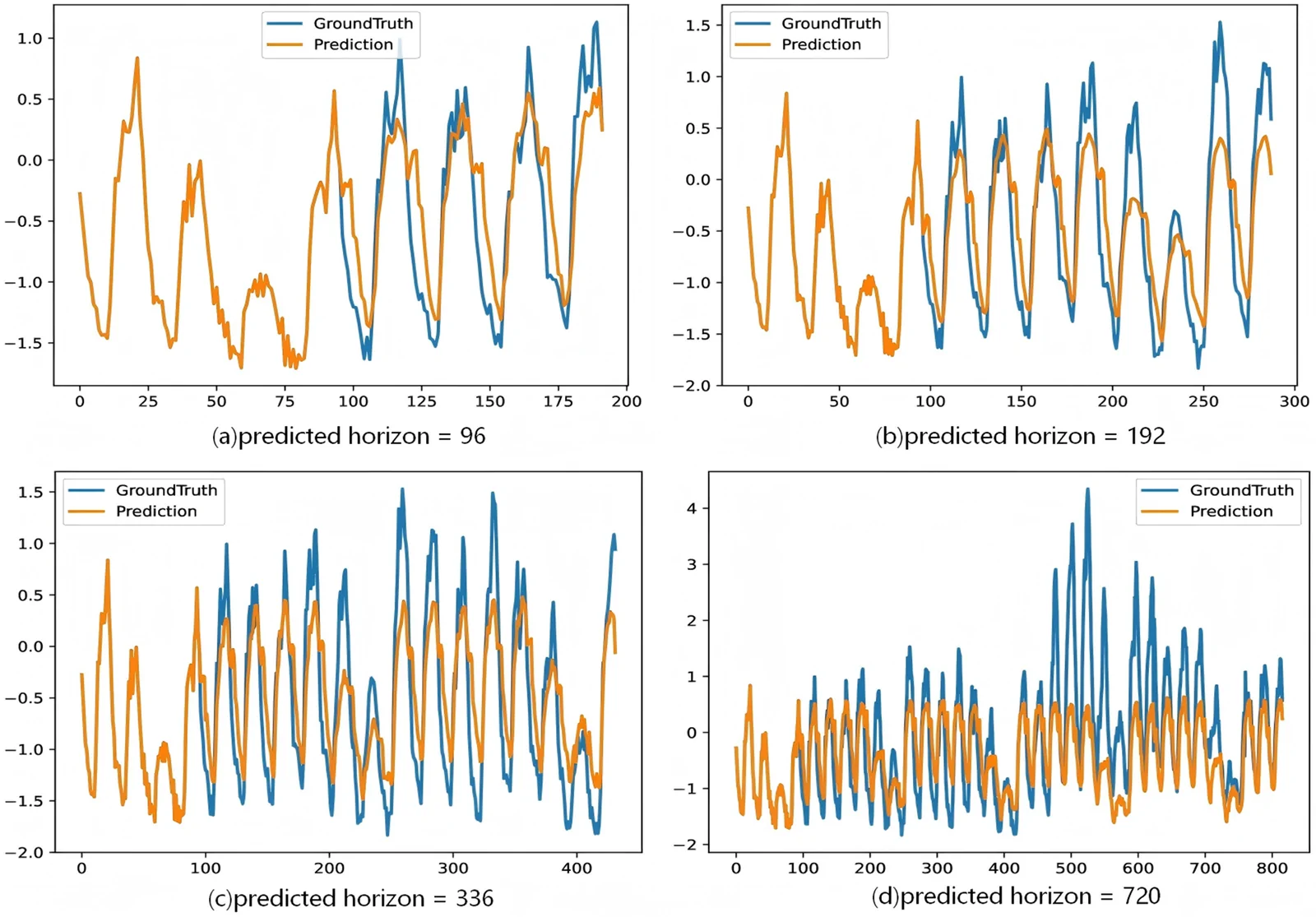
Test results of the four prediction horizons on the ECL dataset. The predicted values (red) and ground truth (blue) are compared across horizons of 96, 192, 336, and 720 time steps. Despite the increase in the prediction horizon, the predicted results maintain an overall trend consistent with the ground truth.

### Robustness and sensitivity analysis

To further assess the stability of PowerNet, we conducted a targeted robustness analysis under two representative settings: ECL with horizon 96 and WTH with horizon 720. These two settings correspond to the ablation study and represent relatively shorter and longer forecasting horizons, respectively. As shown in Table 5, PowerNet maintains stable performance across three independent trials, as reflected by the small standard deviations in both MSE and MAE.

**Table 5:** Robustness analysis under representative forecasting settings.

Dataset	Horizon	MSE	MAE	Trials
ECL	96	0.165±0.003	0.269±0.004	3
WTH	720	0.353±0.006	0.357±0.005	3

We also conducted a lightweight sensitivity analysis on the number of selected dominant periods *k*, which is the key hyperparameter in the FFT-based periodic transformation. As shown in Table 6, PowerNet achieves stable performance when *k* varies within a reasonable range. When *k* is too small, the model may fail to capture sufficient multi-period information, while an excessively large *k* may introduce redundant or noisy periodic components. We further conducted statistical significance tests between PowerNet and the strongest baseline models under the unified experimental protocol. The results show that PowerNet achieves statistically significant improvements in the main comparisons, with p-values below 0.05.

**Table 6:** Sensitivity analysis of the number of selected dominant periods *k.*

Dataset	Horizon	*k*	MSE	MAE	Observation
ECL	96	3	0.172	0.276	
ECL	96	5	0.165	0.269	default
ECL	96	7	0.168	0.272	
WTH	720	3	0.365	0.366	
WTH	720	5	0.353	0.357	default
WTH	720	7	0.359	0.361	

### Ablation study

To verify the effectiveness of the key components within PowerNet, we conducted a targeted component-wise ablation study. The original ablation analysis mainly evaluated the two major branches of the information representation module. To further clarify the contribution of individual components, we expanded the ablation study by separately examining the FFT-based periodic transformation, the Inception variant, the GRU component, and the non-local attention component.

The ablation study was conducted on two representative forecasting settings: the ECL dataset with horizon 96 and the WTH dataset with horizon 720. The former represents a relatively shorter forecasting horizon in electricity load forecasting, while the latter represents a longer forecasting horizon with different temporal characteristics. Using the full PowerNet model as the baseline, we compared it with the following variants: 1) without FFT-based periodic transformation, 2) without the Inception variant, 3) without GRU, 4) without non-local attention, 5) without the complete GRU-NLA branch, and 6) the TimesNet baseline. The results are summarized in Table 7. MSE and MAE are reported in the same order as the comparative experiment table, and the average error increase is calculated relative to the full PowerNet model.

**Table 7:** Metrics for ablation experiment evaluation.

Model Variation	ECL (Horizon 96)		WTH (Horizon 720)		Avg Error Increase
	MSE	MAE	MSE	MAE	
PowerNet	0.165	0.269	0.353	0.357	–
Without FFT-based transformation	0.188	0.295	0.392	0.383	10.49%
Without Inception variant	0.215	0.316	0.356	0.349	11.59%
Without GRU	0.166	0.270	0.361	0.357	0.81%
Without non-local attention	0.167	0.271	0.364	0.358	1.34%
Without GRU-NLA layer	0.167	0.270	0.366	0.358	1.38%
TimesNet (Baseline)	0.168	0.273	0.369	0.361	2.23%

The expanded ablation results provide several observations. First, removing the FFT-based periodic transformation increases the average error by 10.49%, indicating that periodic 2D reconstruction provides useful structured information for long-term forecasting. This result supports the necessity of using frequency-domain information to make intra-period and inter-period dependencies more explicit.

Second, removing the Inception variant leads to an average error increase of 11.59%. The performance degradation is particularly evident on the ECL dataset with horizon 96, suggesting that the multi-scale convolutional branch plays an important role in capturing local periodic structures and short-range cross-period variations. On the WTH dataset with horizon 720, the effect of removing the Inception variant is less pronounced, indicating that local convolutional modeling may be relatively less dominant under longer forecasting horizons.

Third, removing the GRU component or the non-local attention component leads to average error increases of 0.81% and 1.34%, respectively. The larger degradation caused by removing non-local attention suggests that global correlation modeling is beneficial for long-term forecasting, especially when relevant dependencies span multiple periods. Removing the complete GRU-NLA branch results in an average error increase of 1.38%, further confirming that recurrent temporal modeling and non-local dependency modeling provide complementary information to the convolutional branch.

Overall, these ablation results show that PowerNet’s performance improvement is not simply caused by increasing model complexity. Instead, different components contribute to different aspects of temporal representation: the FFT-based transformation provides periodic structure, the Inception variant captures local multi-scale patterns, GRU models sequential transitions, and non-local attention captures global dependencies.

## Conclusion

This study presents PowerNet, a novel long-term power load forecasting model based on multi-periodicity variations and multi-scale feature extraction. To address the challenges of modeling increasingly complex load sequences, the proposed framework leverages the multi-periodic nature of electricity data. By employing the Fast Fourier Transform (FFT) to analyze temporal periodicity, one-dimensional time series are reshaped into a set of two-dimensional feature tensors based on identified dominant periods. This approach effectively introduces image processing concepts into the domain of time series forecasting, treating reconstructed 2D features as spatial-temporal data for enhanced information representation.

The core information representation module utilizes a parallel dual-branch architecture to extract features across multiple scales. The Inception variant branch harnesses the robust spatial feature extraction capabilities of convolutional networks, while the GRU-NLA branch effectively captures long-term dependencies and global correlations within the power data.

The performance of PowerNet was validated through extensive comparative experiments and ablation studies across three public datasets. The results demonstrate that PowerNet achieves superior information representation and significantly lower prediction errors compared to state-of-the-art baseline models. Furthermore, the ablation studies confirm that each integrated component contributes meaningfully to the model’s overall forecasting precision and robustness.

While this study integrates computer vision-inspired techniques with time-series analysis and achieves promising results on the evaluated datasets, the current experimental scope remains limited. The evaluation includes two electricity-related datasets and one weather dataset, which provides useful but not exhaustive evidence for the effectiveness of PowerNet. Therefore, future work will further validate PowerNet on broader long-term forecasting benchmarks and more real-world power load datasets. In addition, we will investigate the integration of more advanced vision-based architectures to further improve forecasting stability and accuracy.
