# Supplementary material for: Long-term electricity load forecasting model based on multi-period and multi-scale analysis
Source: PLoS One. 2026 Oct 30;21(10):e0361106. doi: 10.1371/journal.pone.0361106 (PMC13722309; doi:10.1371/journal.pone.0361106)
Supplement: Peer review history

# Peer review: Decision Letter 0

**DOI:** 10.1371/journal.pone.0361106.r001

**Authors:** Guangyin Jin

14 Apr 2026

Dear Dr. Liu,

Thank you for submitting your manuscript to PLOS ONE. After careful consideration, we feel that it has merit but does not fully meet PLOS ONE’s publication criteria as it currently stands. Therefore, we invite you to submit a revised version of the manuscript that addresses the points raised during the review process.

Please submit your revised manuscript by May 29 2026 11:59PM. If you will need more time than this to complete your revisions, please reply to this message or contact the journal office at plosone@plos.org. When you're ready to submit your revision, log on to https://www.editorialmanager.com/pone/ and select the 'Submissions Needing Revision' folder to locate your manuscript file.

- A letter that responds to each point raised by the academic editor and reviewer(s). You should upload this letter as a separate file labeled 'Response to Reviewers'.
- A marked-up copy of your manuscript that highlights changes made to the original version. You should upload this as a separate file labeled 'Revised Manuscript with Track Changes'.
- An unmarked version of your revised paper without tracked changes. You should upload this as a separate file labeled 'Manuscript'.

If you would like to make changes to your financial disclosure, please include your updated statement in your cover letter. Guidelines for resubmitting your figure files are available below the reviewer comments at the end of this letter.

If applicable, we recommend that you deposit your laboratory protocols in protocols.io to enhance the reproducibility of your results. Protocols.io assigns your protocol its own identifier (DOI) so that it can be cited independently in the future. For instructions see: https://journals.plos.org/plosone/s/submission-guidelines#loc-laboratory-protocols. Additionally, PLOS ONE offers an option for publishing peer-reviewed Lab Protocol articles, which describe protocols hosted on protocols.io. Read more information on sharing protocols at https://plos.org/protocols?utm_medium=editorial-email&utm_source=authorletters&utm_campaign=protocols.

As the corresponding author, your ORCID iD is verified in the submission system and will appear in the published article. PLOS supports the use of ORCID, and we encourage all coauthors to register for an ORCID iD and use it as well. Please encourage your coauthors to verify their ORCID iD within the submission system before final acceptance, as unverified ORCID iDs will not appear in the published article. *Only*  the individual author can complete the verification step; PLOS staff *cannot*  verify ORCID iDs on behalf of authors.

We look forward to receiving your revised manuscript.

Kind regards,

Guangyin Jin

Academic Editor

PLOS One

Journal Requirements:

When submitting your revision, we need you to address these additional requirements.

1. Please ensure that your manuscript meets PLOS ONE's style requirements, including those for file naming. The PLOS ONE style templates can be found at

https://journals.plos.org/plosone/s/file?id=wjVg/PLOSOne_formatting_sample_main_body.pdf and

https://journals.plos.org/plosone/s/file?id=ba62/PLOSOne_formatting_sample_title_authors_affiliations.pdf

2. Please note that PLOS One has specific guidelines on code sharing for submissions in which author-generated code underpins the findings in the manuscript. In these cases, we expect all author-generated code to be made available without restrictions upon publication of the work. Please review our guidelines at https://journals.plos.org/plosone/s/materials-and-software-sharing#loc-sharing-code and ensure that your code is shared in a way that follows best practice and facilitates reproducibility and reuse.

3. Thank you for stating the following financial disclosure:

“National Natural Science Foundation of China (62503028)”

Please state what role the funders took in the study.  If the funders had no role, please state: "The funders had no role in study design, data collection and analysis, decision to publish, or preparation of the manuscript."

If this statement is not correct you must amend it as needed.

Please include this amended Role of Funder statement in your cover letter; we will change the online submission form on your behalf.

4. PLOS requires an ORCID iD for the corresponding author in Editorial Manager on papers submitted after December 6th, 2016. Please ensure that you have an ORCID iD and that it is validated in Editorial Manager. To do this, go to ‘Update my Information’ (in the upper left-hand corner of the main menu), and click on the Fetch/Validate link next to the ORCID field. This will take you to the ORCID site and allow you to create a new iD or authenticate a pre-existing iD in Editorial Manager.

5. Please upload a new copy of Figures 1 - 7 as the detail is not clear. Please follow the link for more information:  https://journals.plos.org/plosone/s/figures

6. If the reviewer comments include a recommendation to cite specific previously published works, please review and evaluate these publications to determine whether they are relevant and should be cited. There is no requirement to cite these works unless the editor has indicated otherwise.

Comments from the Editorial Office:

Please note that reviewer 1 has made only minor comments, and have submitted their review in a language other than English. The editorial office does not require any response from the authors to the comments from reviewer 1 at this time.

[Note: HTML markup is below. Please do not edit.]

Reviewers' comments:

Reviewer's Responses to Questions

**Comments to the Author**

1. Is the manuscript technically sound, and do the data support the conclusions?

Reviewer #1: Yes

Reviewer #2: No

Reviewer #3: Yes

Reviewer #4: Partly

**********

2. Has the statistical analysis been performed appropriately and rigorously?

Reviewer #1: Yes

Reviewer #2: No

Reviewer #3: Yes

Reviewer #4: No

**********

3. Have the authors made all data underlying the findings in their manuscript fully available?

The PLOS Data policy

Reviewer #1: Yes

Reviewer #2: No

Reviewer #3: Yes

Reviewer #4: Yes

**********

4. Is the manuscript presented in an intelligible fashion and written in standard English?

Reviewer #1: Yes

Reviewer #2: Yes

Reviewer #3: Yes

Reviewer #4: Yes

**********

Reviewer #1: 1. 杰出创新

所提出的PowerNet模型创新地结合了FFT频域分析、计算机视觉技术（Inception变体）和时间序列建模（GRU-NLA），为长期负载预测提供了新的技术路径。

2. 全面实验验证

对三个公开数据集（ECL、ETTm1、WTH）进行系统验证，预测视野涵盖96-720个时间步，并与5个主流基线模型进行全面比较，显示了严谨的实验设计。

3. 显著的性能提升

与现有SOTA模型（Autoformer、ETSformer等）相比，PowerNet在MSE和MAE指标上均有显著提升，消融研究也验证了各模块的有效性。

4. 明确的应用价值

长期电力负荷预测对电网规划、发电调度和基础设施建设具有直接指导意义，具有明确的实际应用价值。

5. 强可重复性

使用具有明确数据访问渠道的公开数据集（时间序列库），详细描述模型架构，促进其他研究者的复制和验证。

Reviewer #2: This manuscript proposes PowerNet, a multi-period and multi-scale framework for long-term power load forecasting that integrates FFT-based transformation with CNN, GRU, and attention mechanisms. The topic is relevant and the manuscript is generally well structured. However, there are several concerns regarding novelty, methodological justification, and experimental rigor that need to be addressed.

1. Novelty and positioning

The core idea of transforming time series into 2D representations using frequency-domain analysis and applying computer vision techniques has already been explored in prior work, such as TimesNet. The current manuscript mainly introduces architectural modifications (e.g., an Inception variant and GRU with non-local attention), but it is not clearly demonstrated how these changes constitute a substantial methodological advancement.

The authors should clarify what fundamentally distinguishes PowerNet from existing frequency-based or 2D transformation approaches beyond incremental engineering improvements.

2. Methodological clarity

The FFT-based transformation from 1D time series to 2D tensors is a central component of the model. However, the manuscript does not provide sufficient justification for this design choice. It remains unclear why this transformation preserves or enhances predictive information, and how the selected periodic structures generalize across datasets.

In addition, the interaction among CNN, GRU, and attention mechanisms is not clearly explained. The model appears to combine multiple modules, but the necessity of each component is not well justified.

3. Model complexity and ablation

The model integrates multiple components (CNN, GRU, non-local attention, residual connections), which increases model complexity. However, the manuscript does not include sufficient ablation studies to quantify the contribution of each component.

A detailed ablation analysis is necessary to demonstrate:

the contribution of the FFT-based transformation,

the role of the Inception variant,

the effect of GRU and attention mechanisms.

4. Experimental design and robustness

The reported improvements over baseline models are substantial (e.g., 18%–47% reduction in error), which raises concerns about experimental fairness and robustness.

The manuscript does not report:

results across multiple random seeds,

standard deviation or confidence intervals,

sensitivity to hyperparameters.

Without these analyses, it is difficult to assess the stability of the results.

In addition, it is unclear whether all baseline models were implemented and tuned under consistent settings.

5. Dataset and generalization

The evaluation is conducted on a limited number of datasets (two electricity datasets and one weather dataset). This is not sufficient to support claims of general superiority.

The authors should either include additional datasets or moderate their claims regarding general applicability.

6. Claims on long-term dependency modeling

The manuscript claims improved capability in capturing long-term dependencies. However, no direct evidence or analysis is provided to support this claim.

Additional experiments or analyses are needed to substantiate this statement.

Overall recommendation

The manuscript presents a reasonable engineering approach, but the novelty is limited and the experimental validation is not sufficiently rigorous to support the claimed improvements. Substantial revision is required to strengthen the methodological justification, improve experimental design, and clarify the contribution.

Reviewer #3: This paper presents PowerNet - a deep learning model for long term power load forecasting. Based on a multi-period and multi-scale approach, the model uses FFT to transform one-dimensional time series data into 2D tensors to capture latent periodic patterns. CNN, GRU, and non-local attention are employed to represent long-term dependencies and extract features. Using three public datasets, the authors conducted a comparative evaluation of the proposed model and five baseline models. Experimental results showed that PowerNet generally outperformed the baseline models regarding MSE and MAE. Ablation analysis was conducted to highlight the interpretability of the proposed model and the contribution of each component.

All figures referenced in the manuscript are missing, which negatively impacts the reading and paper evaluation. Please fix the problem and re-submit the manuscript for a second round review.

Overall, the paper is well structured. The model design integrates SOTA technology and demonstrates performance advantage, which can serve as a typical application of deep learning in power load forecasting. The topic should be interesting to certain readers, and the research fits the scope of the PLOS One journal. Other than the figures, no serious technique issues were found in the methodology and results. The literature review can be improved if more related work published in recent two years can be added. An analysis of the pros and cons of the benchmark models can also be added (e.g., as a table) to highlight the novelty of the proposed model.

Reviewer #4: - This paper proposes PowerNet, a long-term prediction model that combines FFT-based periodicity detection and 1D-to-2D reconstruction techniques with a dual-branch representation module consisting of Inception-style expanded convolutional branches and GRU and non-local attention branches.

- While the overall idea is interesting, I believe significant revisions are necessary before it can be considered sufficiently reliable.

- Since the paper states that the baseline results were obtained from the literature, it is unclear whether the baseline models were rerun under the same preprocessing pipeline, hardware, hyperparameter tuning protocol, and random seed as PowerNet.

- Since direct comparison is not possible, the validity of the reported improvements has not yet been fully demonstrated. The baseline models should be rerun under a unified and clearly described experimental environment, or the rationale for the current comparison should be explicitly explained.

- While this paper reports average results from three independent experiments, it does not provide standard deviations, confidence intervals, or statistical significance analyses. This is particularly important given the relatively modest performance observed in the ablation studies.

- In Table 3, the PowerNet values for the 336- and 720-horizon cases on the WTH dataset appear identical; this is an unusual occurrence and requires verification.

- Additionally, the overall model values for WTH (720 hours) reported in Table 4 do not appear to match the corresponding values in Table 3.

- To ensure accuracy, the authors must carefully review all tables and reproduce the results directly from the saved evaluation logs.

- Important implementation details are missing or insufficient, including the exact tensor form after the 1D-to-2D transformation, the method for selecting dominant periods in multivariate data, the hidden dimensions of the GRU, the kernel size and expansion ratio of the Inception variant model, the specifics of inter-branch fusion, the early stopping tolerance, the number of epochs, and the random seed.

- Given that the proposed method relies on the combination of multiple modules, controlled experiments are needed not only on the combined GRU-NLA branches but also on the fusion strategy, the number of dominant periods k, the number of TimesBlocks, and the individual contributions of GRU and non-local attention.

- There are several awkward or grammatically incorrect sentences in the text, and the notation is sometimes difficult to understand; thorough English proofreading is recommended.

**********

what does this mean?). If published, this will include your full peer review and any attached files.

If you choose “no”, your identity will remain anonymous but your review may still be made public.

**Do you want your identity to be public for this peer review?**  For information about this choice, including consent withdrawal, please see our Privacy Policy

Reviewer #1: **Yes:**  biao.cai

Reviewer #2: No

Reviewer #3: No

Reviewer #4: No

**********

[NOTE: If reviewer comments were submitted as an attachment file, they will be attached to this email and accessible via the submission site. Please log into your account, locate the manuscript record, and check for the action link "View Attachments". If this link does not appear, there are no attachment files.]

To ensure your figures meet our technical requirements, please review our figure guidelines: https://journals.plos.org/plosone/s/figures

You may also use PLOS’s free figure tool, NAAS, to help you prepare publication quality figures: https://journals.plos.org/plosone/s/figures#loc-tools-for-figure-preparation.

NAAS will assess whether your figures meet our technical requirements by comparing each figure against our figure specifications.

# Author comment: Author response to Decision Letter 1

**DOI:** 10.1371/journal.pone.0361106.r002

19 Aug 2026

Reviewer #1

Comment 1. Innovative model design

The proposed PowerNet model innovatively combines FFT-based frequency-domain analysis, computer vision techniques represented by an Inception variant, and time-series modeling through GRU-NLA, providing a new technical route for long-term load forecasting.

Response:

We sincerely thank the reviewer for the positive evaluation of the proposed model design. We are pleased that the reviewer recognized the integration of FFT-based periodic analysis, vision-inspired multi-scale feature extraction, and recurrent-attention-based temporal modeling as a meaningful technical route for long-term load forecasting. In the revised manuscript, we further clarified the positioning of PowerNet and emphasized that its contribution lies in enhancing representation learning after periodic 2D reconstruction through the complementary modeling of local multi-scale variations and long-range temporal dependencies.

________________________________________

Comment 2. Comprehensive experimental validation

The manuscript conducts systematic validation on three public datasets, including ECL, ETTm1, and WTH. The prediction horizons cover 96 to 720 time steps, and the proposed model is comprehensively compared with five mainstream baseline models, demonstrating a rigorous experimental design.

Response:

We sincerely thank the reviewer for recognizing the experimental design. In the revised manuscript, we further improved the clarity and reliability of the experimental section. Specifically, we clarified the dataset partitioning, forecasting horizons, baseline comparison protocol, and evaluation metrics. We also added robustness analysis and hyperparameter sensitivity analysis to further support the reliability of the experimental results.

________________________________________

Comment 3. Significant performance improvement

Compared with existing state-of-the-art models such as Autoformer and ETSformer, PowerNet achieves notable improvements in both MSE and MAE. The ablation study also validates the effectiveness of each module.

Response:

We thank the reviewer for this encouraging comment. We are glad that the reviewer recognized the performance advantage of PowerNet and the role of the ablation study. In the revised manuscript, we expanded the ablation analysis to provide a more detailed component-wise evaluation. In addition to the original branch-level ablation, we further examined the contributions of the FFT-based transformation, the Inception variant, the GRU component, and the non-local attention component. These revisions provide clearer evidence that the performance improvement comes from the complementary interaction among periodic reconstruction, multi-scale convolutional modeling, recurrent temporal modeling, and non-local dependency modeling.

________________________________________

Comment 4. Clear application value

Long-term electricity load forecasting has direct practical value for power grid planning, generation scheduling, and infrastructure construction, indicating clear real-world applicability.

Response:

We sincerely thank the reviewer for highlighting the practical significance of this work. We agree that accurate long-term electricity load forecasting is important for power system planning, generation scheduling, load management, and infrastructure development. In the revised manuscript, we retained and strengthened this motivation to better connect the proposed forecasting framework with real-world power system applications.

________________________________________

Comment 5. Strong reproducibility

The study uses public datasets with clear access channels from a time-series library and provides a detailed description of the model architecture, which facilitates replication and validation by other researchers.

Response:

We sincerely thank the reviewer for recognizing the reproducibility of the study. To further improve reproducibility, we revised the manuscript by adding more implementation details, including the tensor form after the 1D-to-2D transformation, the period selection strategy for multivariate data, key architectural settings, training configuration, random seeds, and robustness reporting. These revisions are intended to make the experimental procedure and model implementation clearer for future replication and validation.

Reviewer #2

Comment 1. Novelty and positioning

The reviewer pointed out that the core idea of transforming time series into 2D representations using frequency-domain analysis and applying computer vision techniques has already been explored in prior work such as TimesNet. The reviewer also suggested that the manuscript should clarify what fundamentally distinguishes PowerNet from existing frequency-based or 2D transformation approaches beyond incremental engineering improvements.

Response:

We sincerely thank the reviewer for this important comment. We agree that the frequency-domain-based 1D-to-2D transformation itself has been explored in prior work, especially TimesNet, and should not be presented as the sole novelty of this manuscript. In the revised manuscript, we have therefore clarified the positioning of PowerNet and moderated the related novelty claims.

The key distinction of PowerNet lies not in proposing the 1D-to-2D transformation as a new paradigm, but in designing a task-oriented representation framework for long-term electricity load forecasting after periodic 2D reconstruction. Existing 2D transformation approaches mainly rely on convolutional operations to model local temporal variations in the reconstructed 2D space. While effective, such a design may insufficiently capture long-range temporal dependencies and global correlations that are important for long-term load forecasting. To address this limitation, PowerNet introduces a dual-branch information representation module. The Inception variant branch captures multi-scale intra-period and inter-period variations through dilated convolutional kernels, while the GRU-NLA branch models sequential transitions and non-local temporal correlations. These two branches are integrated through multiplicative feature interaction, allowing local multi-scale features and long-range dependency features to mutually regulate each other.

Accordingly, we have revised the Introduction and Method sections to make the contribution more precise. PowerNet is now positioned as a multi-scale and long-dependency representation framework built upon periodic 2D restructuring, rather than as a purely new frequency-based transformation method. We believe this revised positioning more accurately distinguishes PowerNet from TimesNet and other related 2D transformation approaches.

Modification:

We revised the Introduction, the contribution summary, and the Information Representation Module section. The revised text explicitly states that the novelty of PowerNet lies in the complementary modeling of local multi-scale periodic variations and global long-term dependencies after 2D periodic reconstruction. We also clarified the differences between PowerNet and TimesNet, and avoided overstating the novelty of the FFT-based transformation itself.

________________________________________

Comment 2. Methodological clarity

The reviewer noted that the FFT-based transformation from 1D time series to 2D tensors is a central component of the model, but the manuscript does not provide sufficient justification for this design choice. The reviewer also pointed out that it remains unclear why this transformation preserves or enhances predictive information, how the selected periodic structures generalize across datasets, and how CNN, GRU, and attention mechanisms interact with each other.

Response:

We sincerely thank the reviewer for this insightful comment. We agree that the original manuscript did not provide sufficient methodological explanation for the FFT-based 1D-to-2D transformation and the interaction among the CNN, GRU, and attention components. In the revised manuscript, we have added a clearer justification for both aspects.

First, we clarified why the FFT-based transformation is suitable for long-term load forecasting. Electricity load series usually contain repeated temporal structures caused by daily routines, weekly cycles, seasonal changes, and other periodic consumption behaviors. The FFT-based operation identifies dominant frequency components from the input sequence and converts them into corresponding period lengths. Reshaping the sequence according to these periods does not discard the original temporal information. Instead, it reorganizes the sequence into a structured 2D representation, where one dimension reflects intra-period variations and the other dimension reflects inter-period variations. This makes periodic dependencies more explicit and easier to capture by subsequent feature extraction modules.

Second, we explained how the selected periodic structures can generalize across datasets. The periods are not manually predefined for a specific dataset. They are adaptively selected from the frequency spectrum of each input sequence through a top-k frequency selection strategy. Therefore, PowerNet does not assume a fixed periodic pattern shared by all datasets. Instead, it learns from multiple dominant periods and fuses their representations according to frequency intensities. This adaptive multi-period strategy allows the model to handle different temporal resolutions and periodic characteristics across electricity and weather datasets.

Third, we revised the explanation of the interaction among CNN, GRU, and attention mechanisms. The Inception variant is used to capture local multi-scale patterns in the reconstructed 2D tensor, including short-range intra-period variations and cross-period local structures. The GRU module models sequential transitions and temporal continuity in load evolution. The non-local attention mechanism captures global dependencies between distant temporal positions, especially correlations that cannot be effectively modeled by local convolution alone. These modules are not simply stacked. Instead, the Inception branch and the GRU-NLA branch are organized in parallel and integrated through element-wise multiplication, so that global temporal dependency features can modulate local multi-scale features.

________________________________________

Comment 3. Model complexity and ablation

The reviewer noted that PowerNet integrates multiple components, including CNN, GRU, non-local attention, and residual connections, which increases model complexity. The reviewer also suggested that a more detailed ablation analysis is necessary to quantify the contribution of the FFT-based transformation, the Inception variant, and the GRU and attention mechanisms.

Response:

We sincerely thank the reviewer for this constructive suggestion. We agree that the original ablation study did not sufficiently isolate the contribution of each key component. In the revised manuscript, we have expanded the ablation study in a targeted manner to better evaluate the major design choices of PowerNet while keeping the analysis focused on representative forecasting settings.

Specifically, we retained two representative settings used in the original ablation study: ECL with horizon 96, representing a relatively shorter forecasting horizon in electricity load forecasting, and WTH with horizon 720, representing a longer forecasting horizon with different temporal characteristics. Based on these settings, we added three additional ablation variants: 1) PowerNet without FFT-based periodic transformation, 2) PowerNet without the GRU component, and 3) PowerNet without the non-local attention component. Together with the original variants without the Inception branch and without the complete GRU-NLA branch, these experiments allow us to separately examine the role of periodic 2D reconstruction, multi-scale convolutional modeling, recurrent temporal modeling, and global dependency modeling.

The revised ablation results show that removing the FFT-based transformation leads to an average error increase of 10.49%, indicating that periodic 2D reconstruction provides useful structured information for forecasting. Removing the Inception variant leads to an average error increase of 11.59%, confirming the importance of multi-scale convolutional feature extraction. Removing GRU or non-local attention leads to average error increases of 0.81% and 1.34%, respectively, suggesting that recurrent temporal modeling and global correlation modeling both contribute to forecasting performance, particularly under longer prediction horizons.

We also revised the discussion to avoid overstating the role of any single component. The updated manuscript now emphasizes that PowerNet’s improvement comes from the complementary interaction among periodic transformation, local multi-scale feature extraction, and long-range dependency modeling, rather than from simply increasing the number of modules.

Modification:

We revised the Ablation Study section and expanded Table 4 by adding ablation variants without FFT-based transformation, without GRU, and without non-local attention. We also clarified the interpretation of the ablation results and corrected the reporting order of MSE and MAE to ensure consistency with the comparative experiment table.

________________________________________

Comment 4. Experimental design and robustness

The reviewer noted that the reported improvements over baseline models are substantial, which raises concerns about experimental fairness and robustness. The reviewer also pointed out that the manuscript does not report results across multiple random seeds, standard deviation or confidence intervals, sensitivity to hyperparameters, or whether all baseline models were implemented and tuned under consistent settings.

Response:

We sincerely thank the reviewer for this important comment. We agree that the original manuscript did not provide sufficient information about experimental stability and baseline fairness. In the revised manuscript, we reran the baseline models under a unified experimental protocol and additionally conducted targeted robustness and sensitivity analyses to assess the stability of PowerNet.

First, although the original experiments were repeated over three independent trials, the previous manuscript only reported the average values. In the revised manuscript, we now report mean ± standard deviation for representative settings, including ECL with horizon 96 and WTH with horizon 720. These two settings are consistent with the ablation study and represent relatively shorter and longer forecasting horizons, respectively. The results show that PowerNet maintains stable performance across independent runs, with MSE/MAE values of 0.165 ± 0.003 / 0.269 ± 0.004 on ECL with horizon 96 and 0.353 ± 0.006 / 0.357 ± 0.005 on WTH with horizon 720.

Second, we added a lightweight hyperparameter sensitivity analysis for the most central hyperparameter in the FFT-based transformation, namely the number of selected dominant periods k. Since k directly determines how many periodic structures are retained in the 1D-to-2D reconstruction, this analysis helps assess whether the performance depends heavily on a single manually selected value. The results show that PowerNet remains relatively stable when k varies within a reasonable range. Specifically, on ECL with horizon 96, the MSE/MAE values are 0.172/0.276, 0.165/0.269, and 0.168/0.272 when k is set to 3, 5, and 7, respectively. On WTH with horizon 720, the corresponding values are 0.365/0.366, 0.353/0.357, and 0.359/0.361. These results indicate that the default setting k = 5 achieves the best performance among the tested values, while the model remains reasonably stable when k varies.

Third, we revised the baseline description to clarify the experimental fairness. Specifically, all models were compared under the same dataset splits, input sequence length, prediction horizons, preprocessing procedure, and evaluation metrics. For baseline models, we followed the official implementations or widely used benchmark configurations reported in the corresponding studies, and the recommended hyperparameters were adopted when available. We have revised the ambiguous statement in the original manuscript to avoid the misunderstanding that baseline results were obtained from a different dataset or under inconsistent settings.

Modification:

We revised the Experimental Setup and Comparative Experiments sections. We added the reporting of mean ± standard deviation for representative robustness settings, included a lightweight sensitivity analysis on the number of selected periods k, and clarified the baseline implementation and comparison protocol.

________________________________________

Comment 5. Dataset and generalization

The reviewer noted that the evaluation is conducted on a limited number of datasets, including two electricity-related datasets and one weather dataset. The reviewer suggested that this is not sufficient to support claims of general superiority and recommended either adding additional datasets or moderating the claims regarding general applicability.

Response:

We sincerely thank the reviewer for this valuable comment. We agree that the current evaluation, although covering two electricity-related datasets and one weather dataset, is still limited and should not be used to support overly broad claims of general superiority across all time-series forecasting scenarios.

In the revised manuscript, we have therefore moderated the claims regarding general applicability. Instead of claiming that PowerNet is generally superior across diverse time-series domains, we now state that PowerNet achieves competitive or superior performance on the evaluated datasets and shows preliminary transferability to a non-electricity weather dataset. We also clarify that broader validation on more datasets and application domains is still needed before making stronger claims about general applicability.

Modification:

We revised the Abstract, Comparative Experiments, and Conclusion sections to avoid overgeneralized statements. The revised manuscript now limits the claims to the evaluated datasets and explicitly acknowledges that future work should further validate PowerNet on broader time-series forecasting benchmarks and real-world power load datasets.

________________________________________

Comment 6. Claims on long-term dependency modeling

The reviewer noted that the manuscript claims improved capability in capturing long-term dependencies, but no direct evidence or analysis is provided to support this claim. The reviewer suggested that additional experiments or analyses are needed to substantiate this statement.

Response:

We sincerely thank the reviewer for this valuable comment. We agree that the original manuscript mainly described the long-term dependency modeling capability of PowerNet from the architectural perspective, and the supporting evidence was not sufficiently explained. To address this concern, we provide additional analysis based on the horizon-wise forecasting results and the updated component-wise ablation results.

First, the experimental setting itself includes progressively longer prediction horizons of 96, 192, 336, and 720 time steps. This allows us to examine whether PowerNet maintains stable forecasting performance as the prediction horizon increases. As shown in Table 3, under the longest horizon of 720, PowerNet achieves MSE/MAE values of 0.227/0.322 on ECL, 0.483/0.457 on ETTm1, and 0.353/0.357 on WTH. These results indicate that PowerNet remains competitive under long-horizon forecasting settings, which provides empirical support for its ability to preserve useful temporal information over longer prediction ranges.

Second, the updated ablation results further support the role of the GRU and non-local attention components in long-term dependency modeling. Under the representative long-horizon setting of WTH with horizon 720, removing the GRU component changes the MSE/MAE from 0.353/0.357 to 0.361/0.357, while removing the non-local attention component changes the MSE/MAE to 0.364/0.358. Removing the complete GRU-NLA branch further changes the MSE/MAE to 0.366/0.358. These results suggest that recurrent temporal modeling and non-local correlation modeling contribute to long-horizon forecasting performance. In particular, the larger degradation caused by removing non-local attention indicates that global temporal correlation modeling is beneficial when relevant dependencies span multiple periods.

Therefore, the claim regarding long-term dependency modeling is supported by two types of evidence: horizon-wise forecasting performance across increasing prediction lengths and component-wise ablation under a representative long-horizon setting. We also acknowledge that this evidence is empirical and based on the evaluated datasets, rather than a universal proof of long-term dependency modeling across all possible time-series domains. We have therefore interpreted this claim more cautiously in the response.

Reviewer #3

We sincerely thank the reviewer for the positive and constructive evaluation of our manuscript. We appreciate the reviewer’s recognition that the paper is well structured, that the proposed PowerNet model integrates advanced deep learning techniques for long-term power load forecasting, and that the topic fits the scope of PLOS ONE. We also thank the reviewer for pointing out the missing figures and for suggesting improvements to the literature review and benchmark discussion.

Comment 1. Missing figures

The reviewer pointed out that all figures referenced in the manuscript are missing, which negatively impacts reading and evaluation.

Response:

We sincerely apologize for this issue. The missing figures were caused by an error during manuscript file preparation and conversion. In the revised submission, we have carefully checked the manuscript and reinserted all figures referenced in the text, including the overall architecture of PowerNet, the data processing module, the information representation module, the Inception variant, the non-local attention module, the GRU module, and the prediction visualization results. We have also verified that each figure is correctly numbered, placed, and referenced in the manuscript.

Modification:

We reinserted and checked all figures in the revised manuscript. Specifically, Fig. 1 to Fig. 7 are now included with their corresponding captions and in-text references.

________________________________________

Comment 2. Literature review can be improved by adding more related work published in recent two years

The reviewer suggested improving the literature review by adding more recent studies.

Response:

We thank the reviewer for this helpful suggestion. In the revised manuscript, we have expanded the literature review by incorporating recent studies on long-term time-series forecasting, power load forecasting, and deep learning-based temporal representation learning. These additions better situate PowerNet within recent developments in frequency-domain modeling, Transformer-based forecasting, convolutional temporal modeling, and hybrid deep learning methods for load forecasting.

Modification:

We revised the Introduction section by adding recent related studies published in the last two years and by clarifying how PowerNet differs from existing methods in terms of periodic 2D reconstruction, multi-scale convolutional modeling, and recurrent-attention-based long-range dependency modeling.

________________________________________

Comment 3. Analysis of the pros and cons of benchmark models

The reviewer suggested adding an analysis of the pros and cons of the benchmark models, for example as a table, to highlight the novelty of the proposed model.

Response:

We appreciate this valuable suggestion. In the revised manuscript, we added a comparison table summarizing the strengths and limitations of the benchmark models used in our experiments, including LSTM, Informer, LogTrans, Autoformer, ETSformer, and TimesNet. This table helps clarify the motivation for PowerNet. Specifically, existing models either focus on recurrent temporal modeling, sparse attention, decomposition-based forecasting, or 2D temporal representation. However, they may not jointly model periodic structures, local multi-scale variations, sequential transitions, and global temporal correlations. PowerNet is designed to address this gap through FFT-based periodic reconstruction, a multi-scale Inception variant, and a GRU-NLA branch.

Modification:

We added a new table in the “Baseline models and evaluation metrics” section to compare the benchmark models in terms of their main idea, advantages, limitations, and differences from PowerNet.

________________________________________

We thank the reviewer again for the constructive comments. The revised manuscript has addressed the figure issue, expanded the recent literature review, and added a clearer comparison of benchmark models to strengthen the positioning of PowerNet.

Reviewer #4

Response to Reviewer #4

We sincerely thank the reviewer for the constructive comments and for recognizing that the overall idea of PowerNet is interesting. We agree that the original manuscript did not describe the experimental comparison protocol and robustness analysis clearly enough. In the revised manuscript, we have substantially strengthened the experimental reliability by rerunning the baseline models under a unified experimental setting, reporting uncertainty measures, and adding statistical significance analysis.

Comment 1. Baseline fairness and unified experimental setting

The reviewer noted that the original manuscript stated that the baseline results were obtained from the literature, making it unclear whether the baseline models were rerun under the same preprocessing pipeline, hardware, hyperparameter tuning protocol, and random seeds as PowerNet. The reviewer suggested that the baseline models should be rerun under a unified and clearly described experimental environment, or that the rationale for the current comparison should be explicitly explained.

Response:

We sincerely thank the reviewer for pointing out this important issue. We agree that the original description was ambiguous and could weaken the credibility of the reported improvements. In the revised manuscript, we have clarified this issue and rerun the baseline models under a unified experimental protocol.

Specifically, all baseline models, including LSTM, Informer, LogTrans, Autoformer, and ETSformer, were evaluated using the same preprocessing pipeline, chronological train/validation/test split, input sequence length, prediction horizons, normalization procedure, evaluation metrics, and hardware environment as PowerNet. The experiments were conducted on the same server equipped with an Intel Xeon Platinum 8358P CPU and an NVIDIA GeForce RTX 3090 GPU. For each baseline model, we followed the official implementation or a widely used benchmark implementation, and the hyperparameters were tuned on the validation set or selected according to the recommended settings in the original papers. All models were evaluated under the same prediction horizons of 96, 192, 336, and 720.

This revision ensures that the comparison is a direct head-to-head comparison under a unified experimental environment rather than a comparison against literature-reported results. We also revised the corresponding text in the Comparative Experiments section to make the experimental protocol more transparent.

Modification:

We revised the Comparative Experiments section to clarify that all baseline models were rerun under the same preprocessing pipeline, dataset split, input length, prediction horizons, hardware environment, evaluation metrics, and hyperparameter selection protocol as PowerNet. The previous ambiguous statement about baseline results being obtained from the literature has been removed.

________________________________________

Comment 2. Validity of the reported improvements

The reviewer noted that, if direct comparison is not possible, the validity of the reported improvements has not been fully demonstrated.

Response:

We agree with the reviewer. To address this concern, we reran all baseline models under the same experimental environment and comparison protocol as PowerNet. Therefore, the revised results are based on direct comparisons rather than indirectly collected literature values.

The revised comparison shows that PowerNet achieves competitive or superior performance under the evaluated long-term forecasting settings. We also moderated the wording of the performance claims to avoid overgeneralization. Instead of claiming universal superiority, we now state that PowerNet achieves competitive or superior performance on the evaluated datasets and prediction horizons. This revised wording better reflects the experimental scope and avoids overstating the generality of the results.

Modification:

We revised the result discussion to clarify that the reported improvements are based on unified experimental comparisons. We also moderated claims regarding performance superiority and general applicability.

________________________________________

Comment 3. Standard deviation, confidence interval, and statistical significance

The reviewer noted that although the paper reports average results from three independent experiments, it does not provide standard deviations, confidence intervals, or statistical significance analyses. The reviewer also noted that this is particularly important given the relatively modest performance observed in the ablation studies.

Response:

We sincerely thank the reviewer for this valuable suggestion. We agree that reporting only average values is insufficient for assessing the stability and reliability of the results. In the revised manuscript, we have added uncertainty reporting and statistical significance analysis.

First, all experiments were repeated over three independent trials. In addition to reporting the average results, we now report mean ± standard deviation under representative settings. For example, PowerNet achieves MSE/MAE values of 0.165 ± 0.003 / 0.269 ± 0.004 on ECL with horizon 96, and 0.353 ± 0.006 / 0.357 ± 0.005 on WTH with horizon 720. These small standard deviations indicate that the model maintains stable performance across independent runs.

Second, we calculated confidence intervals for the representative results to further quantify the uncertainty of the reported performance. The confidence interval analysis shows that the performance variation across independent trials remains limited, supporting the robustness of PowerNet.

Third, we conducted statistical significance analysis between PowerNet and the strongest baseline models under the unified experimental protocol. The significance tests show that the improvements of PowerNet are statistically significant under the evaluated settings, with p-values below 0.05. This further supports the reliability of the reported performance gains.

We also revised the ablation discussion to avoid overinterpreting small numerical differences. The revised manuscript now emphasizes that different components contribute to different aspects of temporal representation, including FFT-based periodic reconstruction, multi-scale convolutional modeling, recurrent temporal modeling, and non-local dependency modeling, rather than claiming that every component improves every metric in every setting.

Modification:

We revised the Experimental Setup, Comparative Experiments, Robustness and Sensitivity Analysis, and Ablation Study sections. The revised manuscript now reports mean ± standard deviation, confidence intervals, and statistical significance analysis, and clarifies that the baseline models were rerun under a unified experimental environment.

________________________________________

Comment 4. In Table 3, the PowerNet values for the 336- and 720-horizon cases on the WTH dataset appear identical; this is an unusual occurrence and requires verification. Additionally, the overall model values for WTH (720 hours) reported in Table 4 do not appear to match the corresponding values in Table 3.

Response:

We sincerely thank the reviewer for carefully identifying these inconsistencies. We have rechecked the experimental records and confirmed that this issue was caused by a table-entry error during manuscript preparation.

Specifically, the PowerNet result for the WTH dataset at horizon 720 was mistakenly duplicated from the horizon 336 result in the comparative experiment table. The correct WTH horizon 720 result for PowerNet is MSE = 0.353 and MAE = 0.357. We have corrected this value in the comparative experiment table. Accordingly, the average WTH result of PowerNet has also been recalculated.

We also checked the ablation table and confirmed that the WTH horizon 720 value in the ablation study should be consistent with the corrected comparative experiment result. The revised tables now report the same PowerNet value for WTH horizon 720, namely MSE = 0.353 and MAE = 0.357, ensuring consistency across the comparative experiment and ablation study.

Modification:

We corrected the WTH horizon 720 values of PowerNet in the comparative experiment table and recalculated the corresponding average values. We also checked the ablation table to ensure that the WTH horizon 720 results are consistent across all reported tables.

________________________________________

Comment 5. Important implementation details are missing or insufficient, including the exact tensor form after the 1D-to-2D transformation, the method for selecting dominant periods in multivariate data, the hidden dimensions of the GRU, the kernel size and expansion ratio of the Inception variant model, the specifics of inter-branch fusion, the early stopping tolerance, the number of epochs, and the random seed.

Response:

We sincerely thank the reviewer for pointing out these missing implementation details. We agree that the original manuscript did not provide sufficient information for reproducibility. In the revised manuscript, we have added more detailed descriptions of the implementation settings.

Specifically, we clarified the tensor form after the 1D-to-2D transformation. For each selected period, the input sequence is padded when necessary and reshaped into a 2D tensor, where one dimension represents intra-period variations and the other dimension represents inter-period variations. For multivariate time series, the dominant periods are selected by first computing the FFT amplitude spectrum for each variable and then averaging the amplitudes across variables before selecting the top-k dominant frequencies. This ensures that the selected periods reflect the overall periodic structure of the multivariate input rather than a single variable.

We also added the missing architectural and training details, including the GRU hidden dimension, the kernel size and dilation settings of the Inception variant, the expansion ratio, the inter-branch fusion operation, the early stopping tolerance, the maximum number of epochs, and the random seeds used in the experiments. The inter-branch fusion is implemented through element-wise multiplication between the outputs of the Inception variant branch and the GRU-NLA branch, so that the long-range dependency features can modulate the local multi-scale features.

Modification:

We revised the Data Processing Module, Information Representation Module, and Experimental Setup sections. We also expanded the hyperparameter configuration table to include the missing implementation details required for reproducibility.

________________________________________

Comment 6. Given that the proposed method relies on the combination of multiple modules, controlled experiments are needed not only on the combined GRU-NLA branches but also on the fusion strategy, the number of dominant periods k, the number of TimesBlocks, and the individual contributions of GRU and non-local attention.

Response:

We thank the reviewer for this valuable suggestion. This concern is closely related to the ablation-related comments from Reviewer 2, and we have addressed it by expanding the controlled experiments.

In the revised manuscript, we added component-wise ablation experiments to separately evaluate the contributions of the FFT-based periodic transformation, the Inception variant, the GRU component, the non-local attention component, and the complete GRU-NLA branch. These results show that the improvement of PowerNet does not come from simply adding more modules, but from the complementary interaction among periodic reconstruction, local multi-scale convolutional modeling, recurrent temporal modeling, and non-local dependency modeling.

We also added a sensitivity analysis on the number of dominant periods k, because k is the key hyperparameter controlling how many periodic structures are retained in the FFT-based 1D-to-2D transformation. The sensitivity results show that PowerNet remains stable within a reasonable range of k values, while too few periods may miss important multi-period information and too many periods may introduce redundant or noisy components.

Regarding the fusion strategy and the number of TimesBlocks, we clarified their implementation in the revised manuscript. The default model uses two TimesBlocks with residual connections, and the two branches are fused by element-wise multiplication. This design was selected to balance representation capacity and model complexity while allowing long-range dependency features to modulate local multi-scale features. We have added clearer explanations to avoid the impression that the model is only a simple combination of multiple modules.

Modification:

We revised the Ablation Study and Robustness and Sensitivity Analysis sections. The revised manuscript now includes targeted ablation experiments for the individual contributions of GRU and non-local attention, as well as sensitivity analysis for the number of dominant periods k. We also clarified the fusion strategy and the number of TimesBlocks in the method description.

________________________________________

Comment 7. There are several awkward or grammatically incorrect sentences in the text, and the notation is sometimes difficult to understand; thorough English proofreading is recommended.

Response:

We sincerely thank the reviewer for this suggestion. We carefully proofread the entire manuscript and revised awkward or grammatically incorrect sentences. We also improved the consistency of notation, especially in the descriptions of the 1D-to-2D transformation, the multivariate FFT-based period selection, the GRU-NLA branch, and the ablation study. These revisions make the manuscript easier to read and improve the clarity of the methodological presentation.

Modification:

We conducted a thorough language polishing of the manuscript and revised unclear notation and grammatical issues throughout the text.

Attachment Submitted filename: cover V2.pdf

# Peer review: Decision Letter 1

**DOI:** 10.1371/journal.pone.0361106.r003

**Authors:** Xiaoliang Wei

6 Oct 2026

Long-term electricity load forecasting model based on multi-period and multi-scale analysis

PONE-D-26-09210R1

Dear Dr. Liu,

We’re pleased to inform you that your manuscript has been judged scientifically suitable for publication and will be formally accepted for publication once it meets all outstanding technical requirements.

Within one week, you’ll receive an e-mail detailing the required amendments. When these have been addressed, you’ll receive a formal acceptance letter and your manuscript will be scheduled for publication.

An invoice will be generated when your article is formally accepted. Please note, if your institution has a publishing partnership with PLOS and your article meets the relevant criteria, all or part of your publication costs will be covered. Please make sure your user information is up-to-date by logging into Editorial Manager at Editorial Manager® and clicking the ‘Update My Information' link at the top of the page. For questions related to billing, please contact billing support.

If your institution or institutions have a press office, please notify them about your upcoming paper to help maximize its impact. If they’ll be preparing press materials, please inform our press team as soon as possible -- no later than 48 hours after receiving the formal acceptance. Your manuscript will remain under strict press embargo until 2 pm Eastern Time on the date of publication. For more information, please contact onepress@plos.org.

Kind regards,

Xiaoliang Wei

Academic Editor

PLOS One

Additional Editor Comments (optional):

Reviewers' comments:

Reviewer's Responses to Questions

**Comments to the Author**

Reviewer #3: All comments have been addressed

**********

2. Is the manuscript technically sound, and do the data support the conclusions?

Reviewer #3: Yes

**********

3. Has the statistical analysis been performed appropriately and rigorously?

Reviewer #3: Yes

**********

4. Have the authors made all data underlying the findings in their manuscript fully available?

The PLOS Data policy

Reviewer #3: Yes

**********

5. Is the manuscript presented in an intelligible fashion and written in standard English?

Reviewer #3: Yes

**********

Reviewer #3: The authors addressed the reviewers' comments. The revised manuscript is in a good shape for publication.

**********

what does this mean?). If published, this will include your full peer review and any attached files.

If you choose “no”, your identity will remain anonymous but your review may still be made public.

**Do you want your identity to be public for this peer review?**  For information about this choice, including consent withdrawal, please see our Privacy Policy

Reviewer #3: No

**********

# Editor report: Acceptance letter

**DOI:** 10.1371/journal.pone.0361106.r004

**Authors:** Xiaoliang Wei

PONE-D-26-09210R1

PLOS One

Dear Dr. Liu,

I'm pleased to inform you that your manuscript has been deemed suitable for publication in PLOS One. Congratulations! Your manuscript is now being handed over to our production team.

At this stage, our production department will prepare your paper for publication. This includes ensuring the following:

* All references, tables, and figures are properly cited

* All relevant supporting information is included in the manuscript submission,

* There are no issues that prevent the paper from being properly typeset

You will receive further instructions from the production team, including instructions on how to review your proof when it is ready. Please keep in mind that we are working through a large volume of accepted articles, so please give us a few days to review your paper and let you know the next and final steps.

Lastly, if your institution or institutions have a press office, please let them know about your upcoming paper now to help maximize its impact. If they'll be preparing press materials, please inform our press team within the next 48 hours. Your manuscript will remain under strict press embargo until 2 pm Eastern Time on the date of publication. For more information, please contact onepress@plos.org.

You will receive an invoice from PLOS for your publication fee after your manuscript has reached the completed accept phase. If you receive an email requesting payment before acceptance or for any other service, this may be a phishing scheme. Learn how to identify phishing emails and protect your accounts at https://explore.plos.org/phishing.

If we can help with anything else, please email us at customercare@plos.org.

Thank you for submitting your work to PLOS One and supporting open access.

Kind regards,

PLOS One Editorial Office Staff

on behalf of

Dr. Xiaoliang Wei

Academic Editor

PLOS One
